# Whether and When Superhydrophobic/Superoleophobic Surfaces Are Fingerprint Repellent

**DOI:** 10.34133/2022/9850316

**Published:** 2022-09-23

**Authors:** Chengjiao Wu, Yue Fan, Hongxin Wang, Juan Li, Yuxi Chen, Yingke Wang, Lin Liu, Lidan Zhou, Shilin Huang, Xuelin Tian

**Affiliations:** ^1^Key Laboratory for Polymeric Composite & Functional Materials of Ministry of Education, Guangzhou Key Laboratory of Flexible Electronic Materials and Wearable Devices, School of Materials Science and Engineering, Sun Yat-sen University, Guangzhou 510006, China; ^2^State Key Laboratory of Optoelectronic Materials and Technologies, Sun Yat-sen University, Guangzhou 510006, China; ^3^School of Traditional Chinese Medicine Resources, Guangdong Pharmaceutical University, Guangzhou 510006, China

## Abstract

Driven by the ever-increasing demand for fingerprint-resistant techniques in modern society, numerous researches have proposed to develop innovative antifingerprint coatings based on superhydrophobic/superoleophobic surface design. However, whether superhydrophobic/superoleophobic surfaces have favorable repellency to the microscopic fingerprint is in fact an open question. Here, we establish a reliable method that enables evaluating the antifingerprint capability of various surfaces in a quantitative way. We show that superhydrophobicity is irrelevant with fingerprint repellency. Regarding superoleophobic surfaces, two distinct wetting states of microscopic fingerprint residues, i.e., the “repellent” and the “collapsed” states, are revealed. Only in the “repellent” state, in which the fingerprint residues remain atop surface textures upon being pressed, superoleophobic surfaces can bring about favorable antifingerprint repellency, which correlates positively with their receding contact angles. A finger-deformation-dependent intrusion mechanism is proposed to account for the formation of different fingerprint wetting states. Our findings offer important insights into the mechanism of fingerprint repellency and will help the design of high-performance antifingerprint surfaces for diverse applications.

## 1. Introduction

The pursuit of aesthetic and high-quality life in modern society has been arousing enormous requirements towards antifingerprint coatings in various fields, such as portable electronics [1], automobile interiors [2], protective goggles [3, 4], furniture [5], and packaging [6]. The human fingerprint is a multicomponent mixture and mainly consists of eccrine sweat and sebum with its surface tension in the range of 20-50 mN/m [7–9]. Even though moisture exists in fingerprint liquid, the oily sebum-related components (e.g., fatty acids, wax esters, and glycerides) are generally the main ingredients of fingerprint especially after the volatilization of moisture [10, 11]. To prevent fingerprint liquid from accumulating onto the surface of interest, two typical antifingerprint strategies have been developed to date. The first is the fingerprint-consuming strategy by incorporating photocatalysts or enzymatic biomolecules into porous substrates to degrade fingerprint substances deposited on the surface [12, 13]. However, these approaches have limitations of relying on external energy stimulation and/or slow degradation dynamics. Moreover, due to the complex composition of fingerprint liquids, only specific components could be degraded by the fingerprint-consuming strategy.

Another strategy is to suppress the deposition of fingerprint liquid in the first place by materials with enhanced repellence towards water and oils [2, 5, 7, 8, 14–21]. A variety of researches have deemed that surfaces with high static contact angles (SCAs) to water and oils could repel fingerprint. In particular, superhydrophobic and superoleophobic surfaces with SCAs above 150° are known to have maximized repellency towards water and oil drops by combination of low-surface-energy coatings and roughened surface structures [22–28] and have been put forward for antifingerprint purpose. However, the human fingerprint is a microscopic liquid, and its wetting behaviors on a substrate can be quite different from those of macroscopic drops. In addition, the deposition of fingerprint onto surfaces is different from the deposition of free drops since the former involves external pressing by a fingertip, which further complicates the solid-liquid interface interaction. As a consequence, it remains an open question whether superhydrophobic/superoleophobic surfaces are certainly fingerprint repellent. Surprisingly, despite the extensive reports on antifingerprint surfaces by manipulating their wetting properties, no studies have been devoted to investigate the relationship between surface wettability and fingerprint repellency in a quantitative way, probably due to the lack of reliable assessment methods [12, 29]. Therefore, there is urgent demand to develop reliable methods to quantify the relationship between surface wettability and fingerprint repellency and, furthermore, to uncover whether and when superhydrophobic/superoleophobic surfaces can bring about favorable antifingerprint properties.

Here, we develop a credible method for reliable assessment of the antifingerprint properties of various surfaces with the assistance of artificial fingerprint liquid (AFL) and artificial fingerprint stamp (AFS). Our method consists of quantitative loading of AFL onto AFS with the help of a superoleophilic silicon post surface, deposition of AFL onto the target surface from AFS under certain loads, and accurate weighing of the fingerprint residue using high-precision microbalance with sensitivity down to 0.1 *μ*g. A wide range of samples with varied wetting properties are evaluated with the established method. We found that the water contact angle as well as superhydrophobicity is irrelevant with fingerprint repellency. More importantly, regarding superoloephobic surfaces, favorable fingerprint repellency exists only when the intrusion depth of AFL, which is determined by the deformation degree of AFS upon pressing and the thickness of the AFL film, is less than the microtexture height; in this situation, the antifingerprint performances of the surfaces correlate positively with their receding contact angles. Otherwise, superoleophobic surfaces would deteriorate to a “collapsed” state and lose antifingerprint capability. We envision our findings can advance the development of high-performance antifingerprint materials for diverse applications, such as wearable electronics, smart terminals, touch screen, and optical devices.

## 2. Results and Discussion

**Figure 1:**
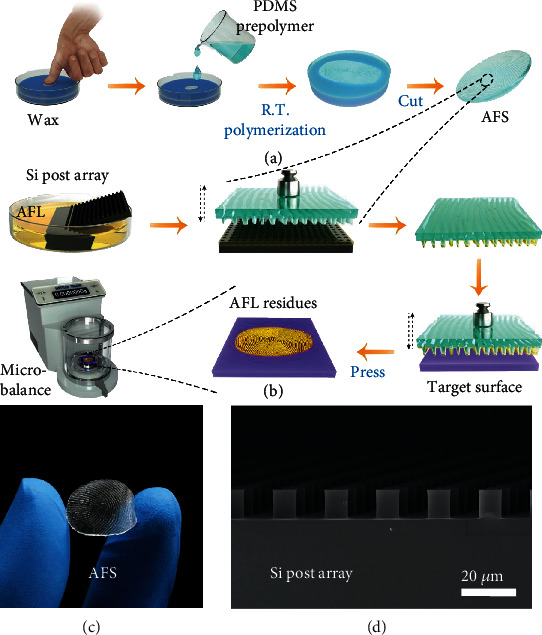
Experimental apparatus and methods for the assessment of antifingerprint property. (a) Schematic showing the preparation of AFS. (b) The process for the assessment of surface antifingerprint property. (c) Photograph of AFS. (d) SEM image of the silicon post surface used to assist AFL transfer.

Our assessment method for fingerprint repellency is illustrated in Figure 1. As the compositions of the human fingerprint vary with a range of factors, such as location, gender, and age [9, 10], AFL with fixed formulation was employed to mimic the real fingerprint to assure repeatable assessments. The essential components of AFL include artificial sweat and sebum-like hydroxylated silicone oil, with 1-methoxy-2-propanol added to enhance their compatibility. The surface tension of AFL is 24.9 ± 1.5 mN/m. This formulation was adopted because it has similar surface tension and viscosity to most real fingerprints [5, 7, 16, 17, 20]. Polydimethylsiloxane (PDMS) elastomer-based AFS was prepared by replication of a human fingertip (Figure 1(a)). PDMS was chosen here because it is an easily accessible and widely used elastomer in soft lithography [1, 4, 6] and has been used to prepare the artificial fingerprint stamp [12]. The monolith of AFS was cut into an elliptic shape (minor axis 2.0 cm and major axis 2.5 cm) to ensure that the whole surface was covered with fingerprint-like patterns. As shown in Figure 1(c), periodically arranged ridges can be clearly seen in AFS. The ridges have widths of 130-190 *μ*m, and the distances between neighboring ridges are about 400-500 *μ*m (Figure S1).

The process of antifingerprint testing consists of quantitative loading of AFL onto AFS, deposition of AFL onto the target surface from AFS under certain loads, and measurement of the weight of fingerprint residue (Figure 1(b)). A superoleophilic silicon surface decorated with arrayed posts (post radius 5 *μ*m, height 11.6 *μ*m, and spacing 20 *μ*m, Figure 1(d)) with an AFL SCA of zero was used to assist the transfer of AFL. The silicon post surface was first brought into contact with a bath of AFL so that a certain amount of AFL was adsorbed to the surface by spontaneous capillary wicking. AFS was then pressed onto the AFL-laden silicon surface under a load of 100 g. Note the size of silicon posts is much less than that of the ridges to facilitate uniform transfer of AFL to AFS. In this way, a fixed quantity of AFL can be loaded onto AFS (Figure S2). The stamp loaded with AFL was subsequently pressed against the target sample under certain loads and released, and the weight of AFL residue on the sample surface was measured using a high-precision microbalance. The applied load was in the range of 25-200 g, which is in accordance with the magnitude of the pressing force exerted by a human on daily subjects, such as pens and smartphones.

**Figure 2:**
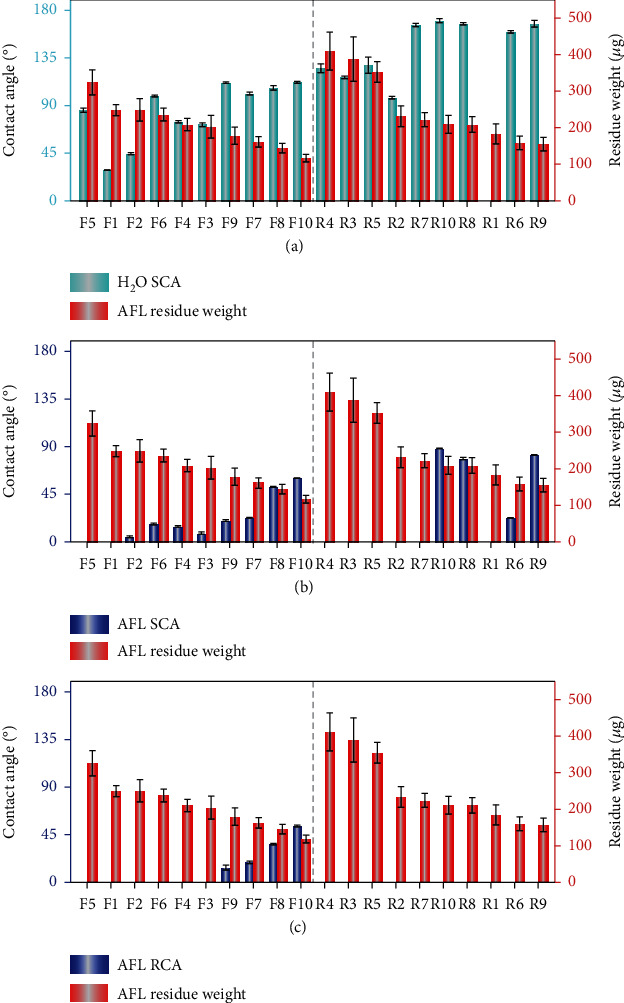
Antifingerprint properties of a range of common flat and rough surfaces. Histograms showing the relationships between AFL residue weight and (a) H_2_O SCAs, (b) AFL SCAs, and (c) AFL RCAs for the investigated samples. The pressing load is 100 g. Details of the samples are listed in Table 1. Note that most of the rough surfaces are superhydrophobic.

To explore the relationship between the antifingerprint repellency and surface wettability, a wide range of easily available samples was investigated, including ten flat surfaces and ten rough surfaces with different structures and surface chemistries (Figure S3, see Experimental for Preparation Details). Their wetting properties, including SCAs, advancing contact angles (ACAs), and receding contact angles (RCAs) towards water and AFL, were thoroughly measured (Table 1). The surface morphologies of these samples were characterized by scanning electronic microscopy (SEM) and atomic force microscopy (AFM, Figure S3). The maximum root-mean square roughness (*R*_q_) values of the flat surfaces are below 2.88 nm, and the structure characteristic sizes of the rough surfaces range from tens of nanometers (Al-boiled) to a few micrometers (PP_rubbed). Most of the rough samples (R6-R10) are superhydrophobic, which allows to access the effect of superhydrophobicity on fingerprint repellency. The antifingerprint properties were evaluated with the established method (Figure 1(b)), in which a load of 100 g was applied when pressing AFS to the target surface. No explicit relationship exists between the weight of AFL residue and water SCAs for either flat or rough samples (Figure 2(a)), suggesting water wettability is in fact irrelevant with fingerprint repellency. The AFL residue weight is also independent of water RCAs (Figure S4). The flat sample F10 is coated with perfluorinated polyether (PFPE), a widely used commercial antifingerprint coating [16, 30, 31], and has a water SCA of about 112°. Notably, all the superhydrophobic surfaces (R6-R10) with water SCAs ranging from 159.7° to 170.4° gave larger AFL residue weight than F10, though the former possessed much higher water repellency. These results indicate clearly that superhydrophobicity cannot assure fingerprint repellency.

We also study the relationship between fingerprint repellency and AFL contact angles. The fingerprint residue weight does not show an explicit dependence on AFL SCAs (Figure 2(b)). However, the fingerprint repellency of flat surfaces appears to increase monotonically with AFL RCAs (Figure 2(c)). This can be reasonably understood. The formation of AFL residue involves the pressing of an AFL liquid film by AFS onto the surface and the subsequent releasing of AFS, in which the liquid film is stretched to a capillary liquid bridge accompanying continuous receding of the contact line. Flat surfaces with larger RCAs facilitate contact line receding and pinch off the liquid bridge at a location closer to the surface, thus leading to the formation of less AFL residue [32, 33]. It should be noted that all the rough samples, including the superhydrophobic ones (R6-R10), are not superoleophobic. AFL liquid would be wicked into surface textures, as confirmed by their vanishing AFL RCAs. As a result, the rough samples exhibit worse fingerprint repellency than the flat surfaces with relatively large AFL RCAs (e.g., F10), even though the former are superhydrophobic.

**Figure 3:**
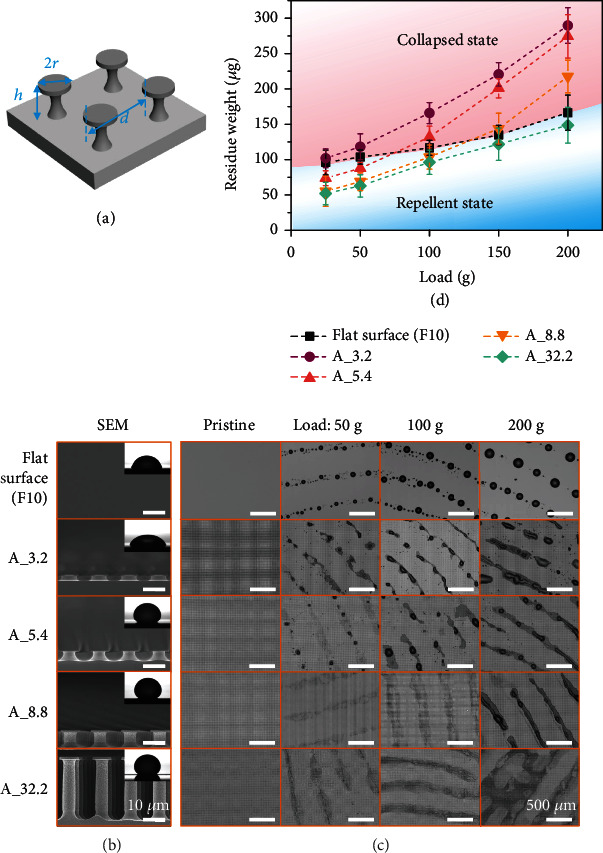
Antifingerprint properties of the microtextured surfaces composed of reentrant posts. (a) Schematic showing the structure parameters of the reentrant silicon posts, in which *h* is the height, *r* the radius of the post cap, and *d* the post-post distance. (b) SEM images of F10 and the prepared reentrant post surfaces. All the samples were coated with PFPE. Insets show the AFL SCAs on the corresponding surfaces. (c) Optical microscope images showing the AFL residues formed on different samples under various pressing loads. (d) The relationship between the AFL residue weight and the load for the samples with different post heights. Typical samples, including A_5.4, A_8.8, and A_32.2, are superoleophobic with similar solid-liquid contact fractions and AFL contact angles. The flat antifingerprint surface F10 is used for comparison.

To further investigate whether superoloephobic surfaces can bring about favorable fingerprint repellency, we prepared a series of microfabricated samples composed of arrayed reentrant silicon posts (Figures 3(a) and 3(b), Figure S5). The samples were modified with PFPE to enhance their liquid repellency (Figure S6 and Table S1) [31]. The reentrant morphology can help to hold oily liquids of low surface tension atop the posts to form a composite solid-liquid-gas interface (i.e., the Cassie-Baxter state), thus facilitating the achievement of superoleophobicity [34–37]. According to the Cassie-Baxter model, the contact angles of the reentrant post surfaces satisfy cos*θ* = *f*_*sl*_(cos*θ*_0_ + 1) − 1, where *θ*_0_ is the intrinsic contact angle of the probe liquid on the flat surface of the same material, and  *f*_*sl*_ is the solid-liquid contact fraction defined as *f*_*sl*_ = *πr*^2^/*d*^2^ (*r* is post radius and *d* spacing) [38]. The surfaces of A_3.2, A_5.4, A_8.8, and A_32.2 have the same post radius and spacing but possess different post heights of 3.2, 5.4, 8.8, and 32.2 *μ*m, respectively (Figure 3(b), Table S2). As expected, the samples of A_5.4, A_8.8, and A_32.2 are superoleophobic and show the same AFL contact angle of around 155° (insets in Figure 3(b)) because of their same solid-liquid contact fraction. A_3.2 does not exhibit superoleophobicity to AFL drops (SCA ~71°), which is probably due to that the saggy liquid-gas interface touches the bottom of the post arrays. We also evaluate the robustness of the superoleophobic surfaces by a robustness factor: RF = *P*_crit_/*P*_ref_, in which *P*_crit_ is the liquid critical breakthrough pressure of the reentrant posts and *P*_ref_ is a characteristic reference pressure which characterizes the minimum pressure exerted by drops on a nonwetting surface [34]. Stable superoleophobicity can only be achieved for surfaces with RF ≫ 1. For the prepared reentrant post surfaces, the breakthrough pressure is expressed as *p*_crit_ = 2*πγr*sin*θ*_0_/(*d*^2^ − *πr*^2^); here, *γ* is the liquid surface tension. The reference pressure *P*_ref_ is equal to 2*γ*/*l*_cap_, where $l_{\text{cap}}=\sqrt{\gamma /\rho g}$ is the capillary length and *ρ* and *g* are liquid density and gravitational acceleration, respectively. For AFL, *l*_cap_ is calculated to be 1.6 mm. Hence, the robustness factor scales as RF = (*πr*sin*θ*_0_/(*d*^2^ − *πr*^2^))*l*_cap_. As shown in Table S2, the prepared reentrant surfaces show AFL breakthrough pressures as high as 4571 Pa and the robustness factors are up to 161, indicating the prepared reentrant surfaces possess robust superoleophobicity towards AFL.

The fingerprint repellency of the reentrant post surfaces was systematically investigated under different loading pressures. The flat antifingerprint coating F10 was used as a reference sample for comparison. At relatively low pressures, light-colored residues were mainly present on the reentrant surfaces (Figure 3(c)). As the pressure increases, considerable dark residues, including AFL droplets with sizes much larger than the post spacings, came into being. The critical pressure for the formation of considerable dark residues varies among different samples and increases with the height of the reentrant posts. While lots of dark residues appeared under a load of 50 g and 100 g for A_3.2 and A_5.4, respectively, the occurrence of dark residues on A_8.8 only took place under a load above 200 g. The weight of AFL residues on the reentrant surfaces under different pressures was measured and compared with that on F10 (Figure 3(d)). The residue weight on A_3.2 is always larger than that on F10, indicating the absence of antifingerprint property. On the contrary, A_32.2 always shows less residues than F10, manifesting excellent fingerprint repellency. Interestingly, A_5.4 and A_8.8 exhibit less residues under low pressures and more residues under increased pressures than F10; i.e., they only show favorable fingerprint repellency at relatively low pressures. The antifingerprint performance of the reentrant surfaces can be indicated by the color shade of the AFL residues: the textured surfaces possess superior antifingerprint capability to F10 in the case of light-colored residues and heavily deteriorated fingerprint repellency once considerable dark residues appear (Figures 3(c) and 3(d)). The above facts suggest the following points: (1) The fingerprint repellency depends on the wetting state of the AFL residues, and the superoleophobic surfaces show favorable antifingerprint properties when the residues are in the “light-colored” state. (2) The wetting state of residues is influenced by the pressures and the texture height, and low pressures and high texture heights facilitate the formation of light-colored residues.

**Figure 4:**
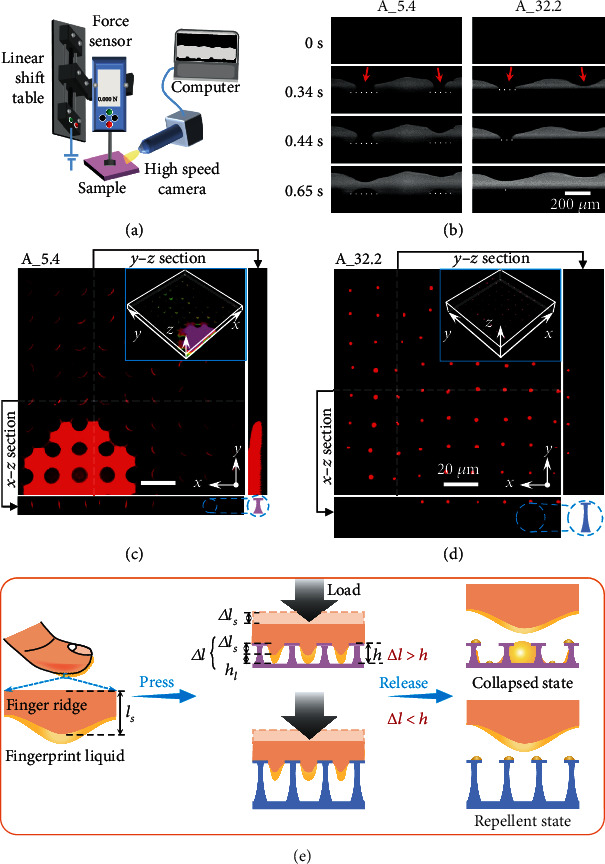
Two distinct wetting states and the corresponding formation mechanism of fingerprint residues on superoleophobic surfaces. (a) Experimental setup for monitoring the dynamic formation process of AFL residues. (b) Sequential images showing the formation of AFL residues on A_5.4 and A_32.2. Red arrows indicate the ridges of AFS. The white dotted lines indicate the contact baselines between the AFL residues and the surfaces. (c, d) CLSM images showing the spatial distribution of fingerprint residues on A_5.4 and A_32.2, respectively. Insets show the three-dimensional profiles. AFL was dyed with Nile red (100 ppm) for fluorescent visualization. The pressing load applied was 200 g. (e) Schematic illustration of the formation mechanism of fingerprint residues.

To reveal the essential difference in the wetting states of the light-colored and the dark AFL residues, we monitored the in situ formation process of residues using a high-speed camera (Figure 4(a)). The AFS stamp was adhered to a force sensor coupled with a linear shift table, which was used to control the pressing of the AFL-laden stamp onto the target sample at given loads. The superoleophobic surfaces of A_5.4 and A_32.2 were selected for comparison of the residue formation under a pressure of 200 g. After pressing, the stamp was lifted at a speed of 0.2 mm/s, and the dynamic formation of AFL residues was recorded by the camera (Figure 4(b) and Movies S1 and S2). Release of the AFS stamp led to the formation of capillary liquid bridges between the AFS ridges and the surface. The continuous stretching and rupture of the capillary bridges gave the occurrence of spherical-cap-shaped AFL residues on A_5.4 and almost invisible residues on A_32.2. Importantly, the contact baselines on A_5.4 kept almost the same during the stretching of the capillary bridge, while continuous and swift contraction of the baselines was observed on A_32.2. That is, the contact line is firmly pinned on A_5.4 and highly mobile on A_32.2. This significant difference strongly suggests that the dark AFL residues on A_5.4 collapsed into the textures. In contrast, the high mobility of the contact line on A_32.2 indicates that AFL kept sitting on the caps of the reentrant posts that facilitated contact line receding and the formation of tiny light-colored residues.

Confocal laser scanning microscopy (CLSM) is a powerful technique to probe solid-liquid interfacial wetting behaviors [37, 39, 40]. Here, CLSM was used to further probe the spatial distribution of the AFL residues on A_5.4 and A_32.2 (Figures 4(c) and 4(d)). A continuous liquid film and meniscus residues can be found from the x-y section image of A_5.4. The y-z and x-z images confirm the AFL residues extended into the textures of A_5.4; that is, the residues are in the “collapsed” state. The continuous residue film was produced just below the AFS ridges where the most concentrated pressure was applied, and the meniscus residues formed at the neighboring area. Optical microscopy investigation was consistent with the above observation (Figure S7). In comparison, only tiny residues are found from the x-y section image of A_32.2, and the y-z and x-z section images verify that the residues remain atop the posts, indicating a “repellent” state to AFL.

Based on the above investigations, we illustrate the two distinct wetting states of AFL residues and their formation mechanism in Figure 4(e). The wetting state is inferred to be determined by the intrusion depth of AFL (Δ*l*) into the texture, which is the sum of the intrusion depth of AFS (Δ*l*_*s*_) and the thickness of the AFL liquid film (*h*_*l*_) adsorbed onto AFS. If the intrusion depth Δ*l* exceeds the texture height, AFL would get collapsed into the structures, and the surfaces lose fingerprint repellency even if they are macroscopically superoleophobic. Favorable fingerprint repellency can only be achieved when Δ*l* is less than the texture height, as observed on reentrant surfaces with high texture heights or under low loading pressures (Figures 3(c) and 3(d)). Upon pressing against the textured surfaces, only a limited area of AFS is in direct contact with the surface and gets compressed due to their nonflat features. The ratio of effective contact area is the product of the solid-liquid contact fraction of the textured surface and the pressure-dependent contact area ratio of AFS (*μ*) onto the surface. As the local compression of AFS results in the intrusion of the uncompressed part into the textures, the intrusion depth of AFS is expected to be determined by the degree of compression deformation at the direct contact area. We can thereby estimate the intrusion depth of AFS with the following equation:

(1) $$\begin{array}{c} ∆l_{s}=\frac{\sigma l_{s}}{Ef_{sl}\mu }, \end{array}$$

where *σ*, *l*_*s*_, and *E* represent the loading pressure, the initial thickness, and the elasticity modulus of AFS, respectively. The total intrusion depth of AFL can then be expressed as

(2) $$\begin{array}{c} ∆l=∆l_{s}+h_{l}=\frac{\sigma l_{s}}{Ef_{sl}\mu }+h_{l}. \end{array}$$

The thickness of the AFL liquid film *h*_*l*_ can be obtained according to the volume of transferred AFL and the actual surface area of AFS. The density and the transferred weight of AFL are about 0.95 g/cm^3^ and 616 *μ*g (Figure S2b), respectively. The actual surface area of AFS is its projection area (3.93 cm^2^) multiplied by the roughness factor (~1.08, evaluated from the cross-section microscopic image of AFS, Figure 4(b)) and is about 4.24 cm^2^. The thickness of the adsorbed AFL film is then calculated to be about 1.5 *μ*m. The initial thickness of AFS was 242 *μ*m, and its elasticity modulus was about 1 MPa (Figure S8). The pressure-dependent contact area ratios of AFS were extracted from the optical microscopic images (Figure 3(c)) using ImageJ, and we can thereby calculate the intrusion depth of AFS under different loading pressures (Table 2).

The calculation results are in well accordance with the experimental observation. The AFS intrusion depth Δ*l*_*s*_ under a load of 50 g is calculated to be 2.3 ± 0.4 *μ*m, and the total intrusion depth of AFL Δ*l* is thus 3.8 ± 0.4 *μ*m. This is larger than the post height of A_3.2 but less than that of A_5.4. As a result, AFL residues mainly remained atop the posts of A_5.4, allowing a favorable fingerprint repellent state for the surface (Figures 3(c) and 3(d)). Under an enhanced load of 100 g, Δ*l*_*s*_ increased to 3.9 ± 0.4 *μ*m, and the corresponding Δ*l* was 5.4 ± 0.4 *μ*m, which reaches the post height of A_5.4 but is below that of A_8.8. Consequently, A_5.4 failed to show fingerprint repellency while A_8.8 still worked. When the load further increased to 200 g, Δ*l*_*s*_ increased to 6.7 ± 1.2 *μ*m, and the corresponding Δ*l* was as large as 8.2 ± 1.2 *μ*m. This intrusion depth is comparable to the post height of A_8.8 and would readily cause collapse of AFL into the texture considering that uneven compression and environmental disturbance could lead to deeper intrusion than expected to a certain extent. Therefore, only A_32.2 with post height far larger than the intrusion depth manifested excellent fingerprint repellency under a load of 200 g.

It should be noted that the collapse of AFL residues reported here is different from the classical Cassie-Baxter to Wenzel transition. In the Cassie-Baxter to Wenzel transition induced by an external pressure [32, 34], the collapse is due to the fact that the liquid hydrostatic pressure overtakes the breakthrough pressure of the microtexture surfaces. In comparison, the collapse of AFL residues is caused by the large deformation of AFS, which drives the intrusion of microscopic AFL liquid films into the microtextures.

Besides AFL, we have also performed the antifingerprint tests on the reentrant post surfaces using a series of artificial sebum (ASB) with different surface tensions and viscosities, which have been used in antifingerprint studies by other researchers. The formulations of the used ASB liquids were listed in Table S3, and their surface tensions and dynamic viscosities are shown in Figure S9. The reentrant surfaces of A_5.4, A_8.8, and A_32.2 exhibited superoleophobic property with SCAs above 150° whereas A_3.2 showed SCA of about 95° to the ASB liquids (Figure S10, a-d). While A_3.2 showed the absence of fingerprint repellency, A_32.2 exhibited excellent antifingerprint property, and the ASB residue weight on A_32.2 was always larger than that on flat F10 under all the investigated pressing loads (Figure S10, e-h). A_5.4 and A_8.8 only manifested fingerprint repellency when the pressing load was lower than certain critical pressure, depending on the texture height. These results are highly similar to the case of AFL and confirm that the model proposed above is applicable to various fingerprint-like liquids.

We also would like to emphasize that though our antifingerprint investigations were conducted with AFL and AFS, the above findings are essential and applicable to practical antifingerprint scenarios. The modulus of the PDMS stamp is comparable to that of real human skin, which was reported to be within 0.46-3.61 MPa [41, 42]. The real-word antifingerprint tests on the reentrant post surfaces show exactly the same wetting behaviors of residues as those observed with AFL and AFS (Figure S11).

**Figure 5:**
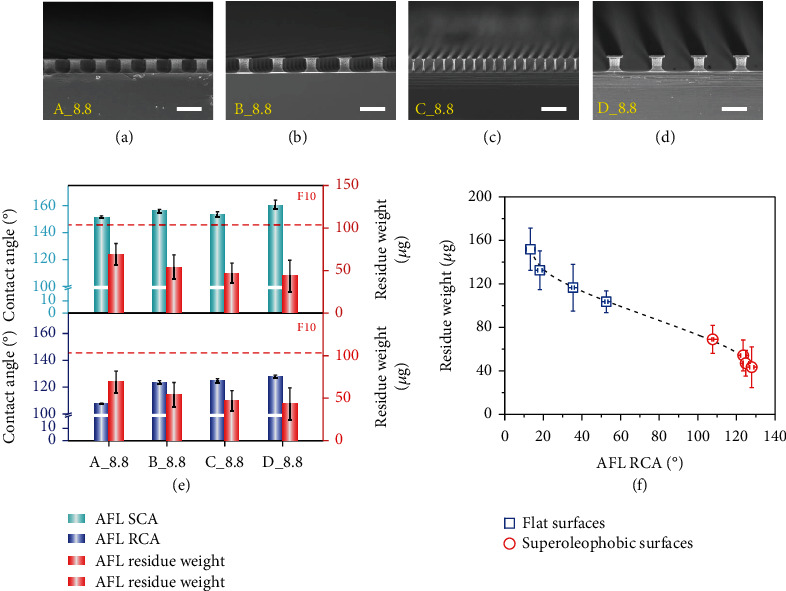
Comparison of the antifingerprint properties of different superoleophobic surfaces working in the fingerprint repellent state. (a–d) SEM images of the prepared samples, namely, A_8.8, B_8.8, C_8.8, and D_8.8. The samples were coated with PFPE and had different solid-liquid contact fractions as well as AFL contact angles. The scale bars are 20 *μ*m. (e) The influence of AFL SCAs (the upper panel) and RCAs (the lower panel) on the residue weight. The red dotted lines indicate the residue weight on F10. (f) Plot showing the relationship between AFL RCAs and the residue weight for the four flat surfaces (F9, F7, F8, and F10) and the four superoleophobic surfaces (A_8.8, B_8.8, C_8.8, and D_8.8). The pressing load was 50 g.

Finally, we investigated how the AFL contact angles influence the fingerprint repellency of superoleophobic surfaces in the repellent state. We prepared a series of superoleophobic reentrant surfaces having different solid-liquid contact fractions but with the same post height of 8.8 *μ*m (Figures 5(a)–5(d)) and measured the weight of AFL residues formed under a pressing load of 50 g. All these surfaces were coated with PFPE and exhibited robust superoleophobicity towards AFL (RF ranging from 40 to 161, Table S4). The intrusion depths of AFL on these surfaces vary from 3.8 ± 0.4 *μ*m to 7.1 ± 0.7 *μ*m (Table S5) as estimated from Equations (1) and (2) and are far below the post height, enabling them to work in the fingerprint repellent state. As expected, all these surfaces showed favorable antifingerprint capability and gave less AFL residues than the flat antifingerprint surface F10. The influence of AFL SCAs and RCAs on the residue weight is shown in Figure 5(e). While the relationship between the residue weight and AFL SCAs is not straightforward, the residue weight decreases monotonically with the RCAs of AFL. We also compared the residue weight for both flat and superoleophobic surfaces (Figure 5(f)). Still, surfaces with larger RCAs showed less AFL residue, and all the superoleophobic surfaces exhibited significantly reduced residue than the flat ones. The above results are understandable as higher AFL RCAs favor rupture of the capillary bridge at a position more adjacent to the surface during liquid film stretching and thus the formation of less residues. Samuel et al. reported that surfaces with RCAs above 90° might enable a liquid drop to be pulled off without residue remaining on the surface, as the capillary bridge was considered to pinch off just on the surface [32]. However, in our experiments, AFL residue was always found to be unavoidable though the superoleophobic surfaces possessed RCAs well above 90°. This is because the capillary bridge would become unstable at the final stage of stretching, which leads to a dynamic RCA far less than the intrinsic RCA accompanying accelerated receding of the contact line. As a result, the capillary bridge would rupture above the surface rather than on the surface, leading to residue formation [33]. Therefore, it is infeasible to completely avoid fingerprint residues. Nevertheless, the AFL RCAs can be used to compare the antifingerprint capability of superoleophobic surfaces working in the fingerprint repellent state.

The reentrant silicon post surfaces were relatively fragile and only used in our study as a model material to investigate the antifingerprint properties of superoleophobic surfaces. However, they still showed certain mechanical durability and could keep intact morphologies after being pressed up to 300 times (Figure S12). We have also demonstrated the application of antifingerprint surfaces in consumer electronics. The superoleophobic surface of A_32.2 was used as a navigation key of a smartphone, which could remarkably reduce the accumulation of the human fingerprint compared with the original navigation key and thus improve the key's cleanliness and aesthetic (Figure S13).

## 3. Conclusion

In summary, we have developed a reliable evaluation method with the assistance of AFL and AFS that could be used for standardized antifingerprint testing of various surfaces. We show that the water contact angle is an irrelevant factor and superhydrophobicity alone has nothing to do with fingerprint repellency. As for superoleophobic surfaces, we reveal two distinct wetting states of fingerprint residues, namely, the “repellent” and the “collapsed” states, which are dictated by the deformation of AFS and the total intrusion depth of AFL into surface textures. Favorable fingerprint repellency can only be achieved for superoleophobic surfaces when the intrusion depth is less than the texture height; otherwise, the surfaces would give deteriorated antifingerprint properties even worse than that of flat antifingerprint surfaces. We also demonstrate that the antifingerprint capability of superoloephobic surfaces working in the repellent states can be optimized by enhancing their AFL RCAs. Our study is fundamentally important to understand the mechanism of fingerprint repellency of textured surfaces, which helps not only to clarify whether and when superhydrophobic/superoleophobic surfaces bring about favorable fingerprint repellency but also to develop high-performance antifingerprint surfaces for diverse applications.

## Figures and Tables

**Table 1:** The water and AFL contact angles of the flat and rough samples with varied surface wettability. The samples are numbered according to their water SCAs.

Samples		Designations	Water contact angles (°)			AFL contact angles (°)		
			SCA	ACA	RCA	SCA	ACA	RCA
Flat samples	Glass	F1	29.0 ± 0.1	36.6 ± 0.6	5.4 ± 1.4	0	0	—
	Glass-PDA	F2	44.5 ± 1.0	51.1 ± 0.5	16.4 ± 0.4	4.7 ± 0.8	6.4 ± 1.1	—
	PET	F3	72.2 ± 1.7	74.9 ± 0.4	45.5 ± 0.9	8.3 ± 1.0	10.2 ± 1.3	—
	Glass-OTS	F4	74.7 ± 1.1	94.6 ± 1.2	63.5 ± 1.7	14.5 ± 0.7	17.6 ± 2.3	—
	PS	F5	85.6 ± 2.0	95.4 ± 0.7	71.4 ± 2.9	0	0	—
	PP	F6	99.1 ± 0.8	107.9 ± 1.0	90.4 ± 0.6	16.9 ± 0.8	18.7 ± 5.1	—
	Glass-LPDMS	F7	101.4 ± 1.2	106.3 ± 0.5	95.0 ± 0.4	22.8 ± 0.4	29.7 ± 1.0	18.2 ± 1.2
	Glass-PFOS	F8	106.7 ± 2.1	111.4 ± 0.4	92.9 ± 1.2	51.9 ± 0.5	58.2 ± 0.5	35.4 ± 0.7
	Glass-CPDMS	F9	111.7 ± 0.8	121.7 ± 0.3	101.3 ± 0.3	20.0 ± 0.8	32.3 ± 1.9	13.3 ± 2.3
	Glass-PFPE	F10	112.0 ± 1.0	119.2 ± 0.5	107.5 ± 0.4	60.2 ± 0.2	64.3 ± 1.0	52.6 ± 0.7

Rough samples	Al-boiled	R1	0	0	—	0	0	—
	PE_rubbed	R2	97.5 ± 1.1	124.1 ± 1.1	42.5 ± 3.3	0	0	—
	Frosted glass	R3	116.7 ± 1.1	—	—	0	0	—
	PS_rubbed	R4	125.3 ± 4.2	132.4 ± 0.7	33.2 ± 4.7	0	0	—
	PP_rubbed	R5	128.1 ± 7.8	146.2 ± 0.8	78.6 ± 2.3	0	0	—
	Al-boiled-PFPE	R6	159.7 ± 1.2	164.5 ± 2.5	102.9 ± 1.2	22.6 ± 0.2	26.2 ± 1.5	—
	Never wet	R7	166.3 ± 1.5	167.9 ± 2.6	158.3 ± 1.7	0	0	—
	Frosted glass-PFPE	R8	167.3 ± 1.1	169.6 ± 0.5	140.0 ± 1.6	78.7 ± 1.1	83.2 ± 2.3	—
	BS-PFPE	R9	167.3 ± 3.2	169.4 ± 0.5	158.9 ± 1.1	82.1 ± 0.3	83.7 ± 1.5	—
	BS-PFOS	R10	170.4 ± 1.9	174.6 ± 1.3	163.1 ± 2.4	88.3 ± 0.2	100.6 ± 1.8	—

**Table 2:** Calculation of the intrusion depth of AFS under different pressures.

Loading weight	Pressure (*σ*)	Solid-liquid contact fraction (*f*_*sl*_)	Pressure-dependent contact area ratio (*μ*)	AFS intrusion depth (Δ*l*_*s*_)
50 g	1247 Pa	0.35	0.38 ± 0.07	2.3 ± 0.4 *μ*m
100 g	2494 Pa	0.35	0.44 ± 0.04	3.9 ± 0.4 *μ*m
200 g	4988 Pa	0.35	0.52 ± 0.09	6.7 ± 1.2 *μ*m

## Data Availability

The data used to support the findings of this study are present in the paper and the supporting materials.
