# Supplementary material for: Whether and When Superhydrophobic/Superoleophobic Surfaces Are Fingerprint Repellent
Source: Research (Wash D C). 2022 Sep 23;2022:9850316. doi: 10.34133/2022/9850316 (PMC9534580; doi:10.34133/2022/9850316)
Supplement: Supplementary Materials — Supplementary information is available at https://doi.org/. Experimental details and extra characterizations are found in Figures S1–S13, Tables S1–S6, and Movies S1–S2. [file 9850316.f1.zip › Revised Supporting Information (Clean Version).docx]

Supplementary Materials for

**Title**

**Whether** **and when superhydrophobic/superoleophobic surfaces are fingerprint repellent**

**Short titles: Fingerprint repellency of super-liquid-repellent surfaces**

**Authors**

Chengjiao Wu,^1,2^ Yue Fan,^1,2^ Hongxin Wang,^1,2^ Juan Li,^1,2,3^ Yuxi Chen,^1,2^ Yingke Wang,^1,2^ Lin Liu,^2^ Lidan Zhou,^2^ Shilin Huang,^1,2^ Xuelin Tian^1,2,*^

**Affiliations**

^1^Key Laboratory for Polymeric Composite & Functional Materials of Ministry of Education, Guangzhou Key Laboratory of Flexible Electronic Materials and Wearable Devices, School of Materials Science and Engineering, Sun Yat-sen University, Guangzhou 510006, China

^2^State Key Laboratory of Optoelectronic Materials and Technologies, Sun Yat-sen University, Guangzhou 510006, China

^3^School of Traditional Chinese Medicine Resources, Guangdong Pharmaceutical University, Guangzhou 510006, China

*Corresponding author. E-mail: tianxuelin@mail.sysu.edu.cn (X. Tian)

**Materials and Methods**

**Preparation of artificial fingerprint stamp (AFS).**

The fabrication of AFS is schematically shown in Figure 1a. A certain amount of paraffin wax was melted in a glass petri dish, and the right index finger of a volunteer (male, age 31) was put onto the melting wax for a few seconds while the wax was cooled down simultaneously. The wax layer then served as a negative mold, and a mixture of PDMS prepolymer (Sylgard 184 elastomer kit and curing agent, weight ratio 10:1, Dow corning) was poured onto the negative mold and then cured at room temperature for 24 h. AFS was obtained by peeling off the cured PDMS elastomer and customizing it to an ellipse with a minor axis of 2 cm and a major axis of 2.5 cm.

**Preparation of artificial fingerprint liquid (AFL) and artificial sebum (ASB).**

AFL was prepared according to a previously-reported recipe [1-5]. First, 3 mL of lactic acid (98%, Macklin), 5 mL of acetic acid (AR, 99.5%, Macklin), 10 g of sodium chloride (AR, 99.5%, Macklin), 10 g of sodium hydrogen phosphate (99%, Macklin) and 1 L of deionized water were mixed and stirred for 1 h to generate the artificial sweat. It was then mixed with 1-methoxy-2-propanol (99%, Macklin) and hydroxy-group-terminated polydimethylsiloxane (PDMS-OH, *M*n ~ 550, viscosity ~ 25 cSt, Sigma-Aldrich) with a weight ratio of 3:1:1. Finally, AFL was obtained after 1 h ultrasonic processing and 1 h quiescence before use.

ASB-1 was supplied by Shenzhen Zhongwei Equipment Co., Ltd. ASB-2, ASB-3 and ASB-4 were prepared according to the formulations listed Table S3.

**Surface preparation.**

The surfaces in Table 1 and Figure 2 (F1 to F10, R1 to R10) are prepared as follows:

F1 (glass sample) was a glass slide obtained from Shanghai Titan Scientific Co., Ltd.

F2 (glass-PDA sample) was a glass slide coated with polydopamine. It was prepared by dip coating a glass slide in a tris-buffer (pH 8.5) solution of dopamine (98%, Sigma-Aldrich) and polymerizing for 6 h.

F3 (PET sample) was a flat polyethylene terephthalate sheet purchased in local market.

F4 (glass-OTS sample) was a glass slide coated with *n*-octadecyltrichlorosilane (OTS, 95%, Alfa Aesar). It was obtained by immersing an oxygen plasma-treated glass slide into 0.1 vol% OTS solution in toluene for 2 h.

F5 (PS sample) was a flat polystyrene sheet purchased in local market.

F6 (PP sample) was a flat polypropylene sheet purchased in local market.

F7 (glass-LPDMS sample) was a glass slide covalently tethered with linear polydimethylsiloxane brushes. It was prepared by plasma treatment of the glass slide followed with annealing at 100 °C in silicone oil (*M*n ∼ 6000, Alfa Aesar) for 24 h.

F8 (glass-PFOS sample) was a glass slide coated with 1*H*,1*H*,2*H*,2*H*-perfluorooctyltrichlorosilane (PFOS, 97%, Sigma-Aldrich). It was prepared via a facile chemical vapor deposition (CVD) method by placing a plasma-treated glass slide with 20 μL PFOS in a desiccator and the CVD process lasted for 2 h.

F9 (glass-CPDMS sample) was a glass slide coated with crosslinked PDMS. First, a plasma-treated glass slide was immersed into 0.1 wt% mixture of Sylgard 184 elastomer kit and curing agent (ratio 10:1) in *n*-hexane for 30 min. It was then taken out and the prepolymers on the surface was further cured at 80 °C for 2 h.

F10 (glass-PFPE sample) was a glass slide covalently tethered with perfluorinated polyetherterminated trimethoxysilyl (PFPE, *Optool* UD509, Daikin). It was prepared by dip coating a glass slide in PFPE solution (0.4 wt%) followed with annealing at 130 °C for 30 min in an oven.

R1 (Al-boiled sample) was an aluminum foil boiled in water of 90 °C for 2 h.

R2 (PE_rubbed sample) was a polyethylene sheet abraded by a 2000 mesh sandpaper for 10 cycles.

R3 (frosted glass sample) was obtained from the frosted area of a glass slide.

R4 (PS_rubbed sample) was a PS sheet abraded by a 2000 mesh sandpaper for 10 cycles.

R5 (PP_rubbed sample) was a polypropylene sheet abraded by a 2000 mesh sandpaper for 10 cycles.

R6 (Al-boiled-PFPE sample) was a boiled aluminum foil coated with PFPE by the aforementioned dip-coating method.

R7 (NeverWet sample) was a glass slide coated with a commercial NeverWet (Rust-Oleum) film by spray coating.

R8 (frosted glass-PFPE sample) was a frosted glass coated with PFPE by the abovementioned aforementioned dip-coating method.

R9 (BS-PFPE sample) was a black silicon surface coated with PFPE. The black silicon was prepared by reactive ion etching (RIE, Oxford Instruments, PlasmaPro System 100RIE, radio frequency power = 100 W, SF_6_ flow rate = 15 sccm, O_2_ flow rate = 15 sccm, pressure = 50 mTorr, etching time = 15 min). The PFPE coating was modified on the black silicon through the aforementioned dip-coating method.

R10 (BS-PFOS sample) was a black silicon surface coated with PFOS. It was obtained via the aforementioned CVD method.

**Preparation of straight silicon post array.**

A micro-arrayed soft mask of photoresist AZ nLOF 2035 (MicroChemicals) was first generated on the silicon substrate by photolithography. After development and rinse, the surface was etched using deep reactive ion etching (DRIE, Oxford Instruments, PlasmaPro 100 Estrelas, ICP forward power = 1000 W, SF_6_ flow rate = 65 sccm, C_4_F_8_ flow rate = 50 sccm, pressure = 8.4 mTorr, etching time = 10 min). Ultimately, the micro-structured surface with post array was generated by a 5 min O_2_ plasma treatment to remove the remaining photoresist.

**Preparation of reentrant silicon post arrays.**

The fabrication process of the reentrant posts is illustrated in Figure S5. A silicon wafer with 1 μm silicon dioxide top was patterned with micro-arrayed soft mask of photoresist AZ nLOF 2035 by photolithography. The wafer was treated by RIE for SiO_2_ etching (radio frequency power = 200 W, CHF_3_ flow rate = 12 sccm, Ar flow rate = 38 sccm, pressure = 30 mTorr, etching time = 22 min). The posts were generated through two different methods for different requirements of the heights. To obtain relatively high posts, the Bosch process of varied loops (deposition step for 1.5 s, etch step 1 for 1.5 s, etch step 2 for 1.5 s for one loop) was utilized by DRIE. For relatively short post, an anisotropic etching was used by inductively coupled plasma (ICP, Oxford Instruments, PlasmaPro System 100ICP180, ICP forward power = 800 W, SF_6_ flow rate = 30 sccm, O_2_ flow rate = 15 sccm, pressure = 6 mTorr, temperature = 10 °C). Finally, the reentrant topography was achieved by an isotropic silicon etching using ICP (ICP forward power = 500 W, SF_6_ flow rate = 30 sccm, O_2_ flow rate = 10 sccm, pressure = 6 mTorr, temperature = 25 °C). All samples were O_2_ plasma cleaned for 5 min. Noted that the reentrant surfaces with various post heights used in this study were prepared following the process parameters (DRIE etching loops and ICP etching time) listed in Table S6. The reentrant surfaces were modified with PFPE using the aforementioned dip-coating method to render them superoleophobicity.

**Characterization.**

Optical microscopic images were recorded by an optical microscope (Leica DM2700M). Three-dimensional microscopic images were observed by a stereoscopic microscope (Leica DVM6). Scanning electron microscopy (SEM) images were observed by a focused ion beam equipped SEM system (FIB-SEM, Zeiss Auriga). Atomic force microscope (AFM) images were recorded using Icon Dimension (Bruker). The NanoScope Analysis software was used to calculate the root-mean-square roughness (*R*_q_) of AFM images. Surface chemistries of samples were analyzed by X-ray photoelectron spectroscopy (XPS, ESCALAB 250, Thermo Fisher Scientific) with a monochromatized Al K*α* X-ray source (1486.6 eV). The adventitious containment carbon at C 1s = 284.8 eV was used to calibrate all binding energies. Contact angle measurements were performed on a goniometer DSA 100S (KRÜSS GmbH). For the ACAs and RCAs tests, the needle-in-the-sessile-drop method (0.05 μL/s injection and suction speed) was used for flat samples, while the tilting plate method (10 °/min tilting speed) was utilized for textured samples. The ACAs were measured using 5 μL water and AFL, while the RCAs were measured using 20 μL water and 5 μL AFL. All the contact angle values were tested at least three times at different positions. Liquid surface tension was examined by pendant-drop method on DSA 100S. The dynamic viscosity measurement of AFL and ASB was performed on a MCR 302 rheometer (Anton Paar GmbH) with a parallel-plate fixture (25 mm diameter) in a dynamic frequency sweep mode. The fluorescence images of fingerprint residues were observed by confocal laser scanning microscopy (CLSM, Nikon C2+ system). The tensile strain-stress curve of AFS was measured with a universal testing machine (HZ-1007C, Dongguan Lixian Co., Ltd.).

**Anti-fingerprint property testing.**

A credible process is established to analyze the residual weight of AFL on the surfaces. First, a silicon post surface (post radius 5 μm, height 11.6 μm, and spacing 20 μm) was first brought into contact with a bath of AFL for 15 min so that an AFL liquid film was adsorbed to the surface by spontaneous capillary wicking. AFS was then pressed onto the AFL-laden silicon post surface under a 100 g load for 10 s to imitate an oily “finger skin”. AFS loaded with AFL was subsequently pressed against the target sample under certain loads for 10 s and released. To obtain the final net weight of AFL residues, an analytical micro-balance (Mettler-Toledo XPR2U) with sensitivity down to 0.1 μg was used to measure the weight of the sample before and after AFS pressing. A static electricity eliminator was equipped to eliminate the effect of surface charge and enable accurate weighing.

**Observation of dynamic formation of AFL residues.**

The dynamic generations of AFL residues on the selected A_5.4 and A_32.2 samples were recorded by a high-speed camera (VEO 710L, Phantom). A linear shift table (SL40E150-10C7, Chengdu Fuyu Co., Ltd.) combined with a force sensor (SH-200, Leiqing Hanpi Co., Ltd.) was used to control the pressing of the AFL-laden AFS onto the samples at given loads. The subsequent lifting of AFS was controlled by the linear shift table at a uniform speed of 0.2 mm/s.

**Demonstration** **of practical application of anti-fingerprint.**

A A_32.2 sample was placed on the navigation key of a smartphone (provided by a confidential merchant). The residues on original and A_32.2 loaded navigation key were produced by pressing the right index finger of an adult male under a pressure of about 100 g for 10 s. Then they were recorded by a digital camera (AO-HD228S, Shenzhen Aosvi Optical Instrument Co., Ltd).


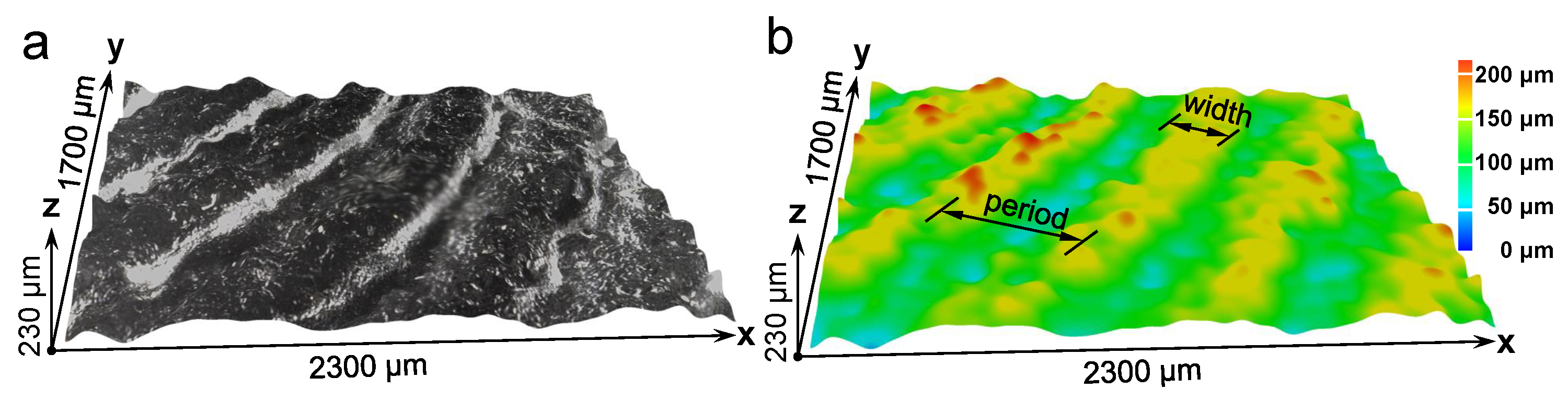


**Figure S1**. (a) Three-dimensional image and (b) the corresponding topographic map of AFS photographed by a stereoscopic microscope.


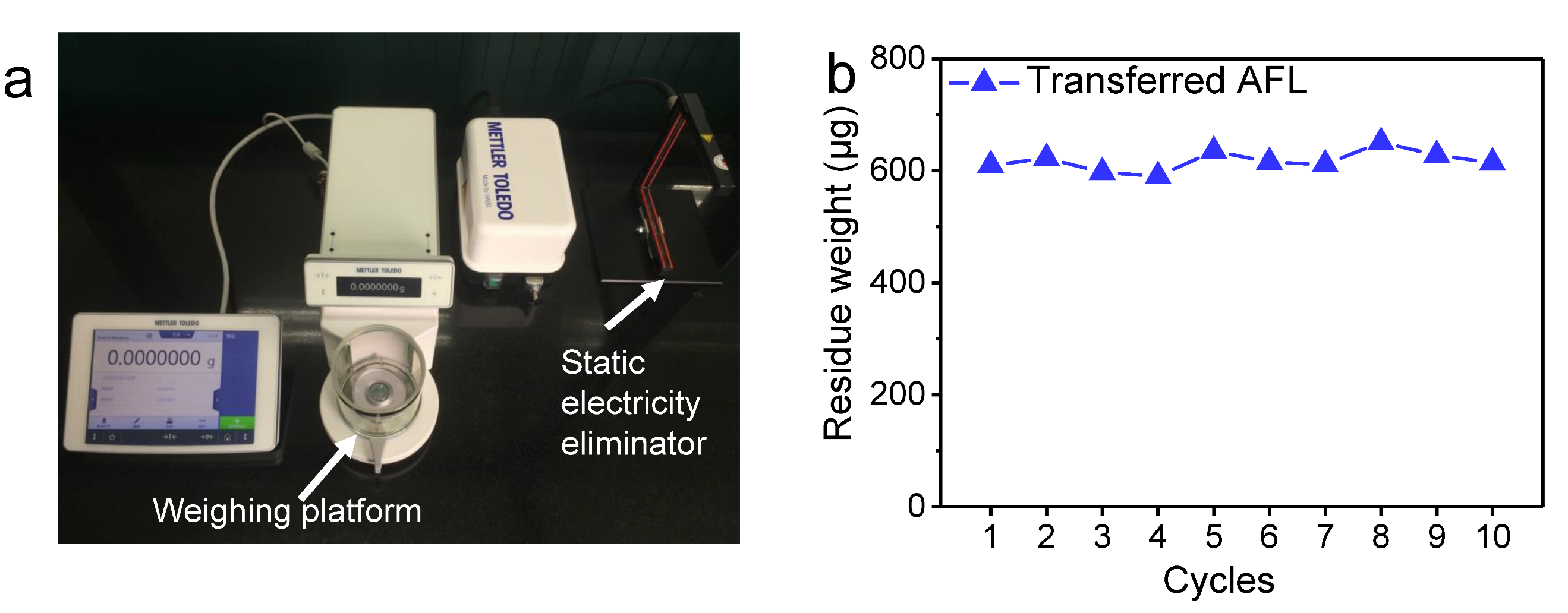


**Figure S2**. (a) The high-precision micro-balance used for weighing in this study. The balance has a sensitivity down to 0.1 µg, and a static electricity eliminator was used to avoid the effect of electrostatic interaction on weighing. (b) Repeated AFL transferring tests confirmed that AFL can be quantitatively transferred to AFS. The weight of AFL transferred to AFS was 616 ± 18 μg.


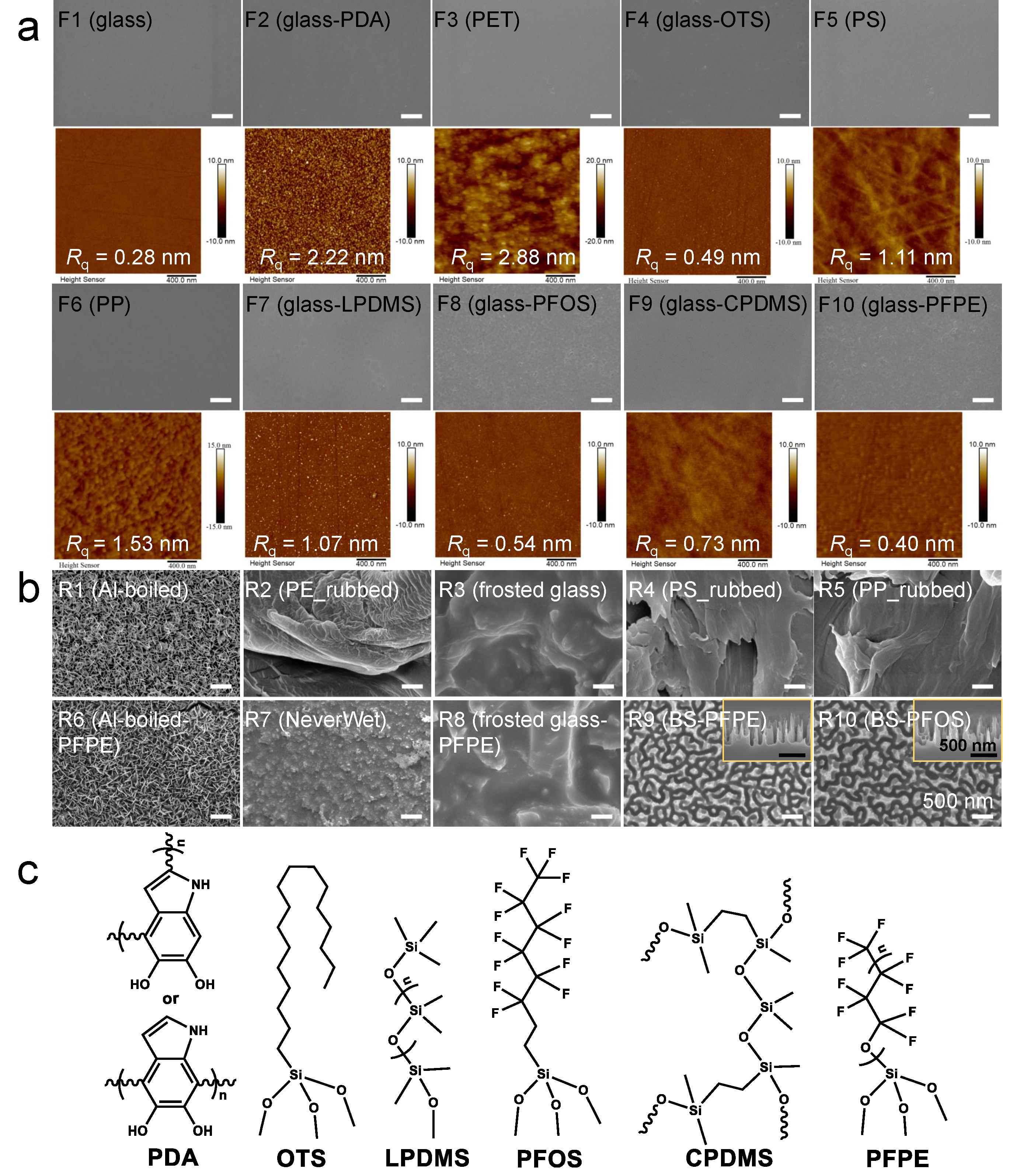


**Figure S3**. (a) SEM and AFM images of the ten flat samples (F1-F10). (b) SEM images of the ten rough samples (R1-R10). The insets in the last two images show the side-view morphologies of the respective samples. (c) The structural formulas of the chemical compounds used for surface modification are listed, including PDA, OTS, LPDMS, PFOS, CPDMS and PFPE.


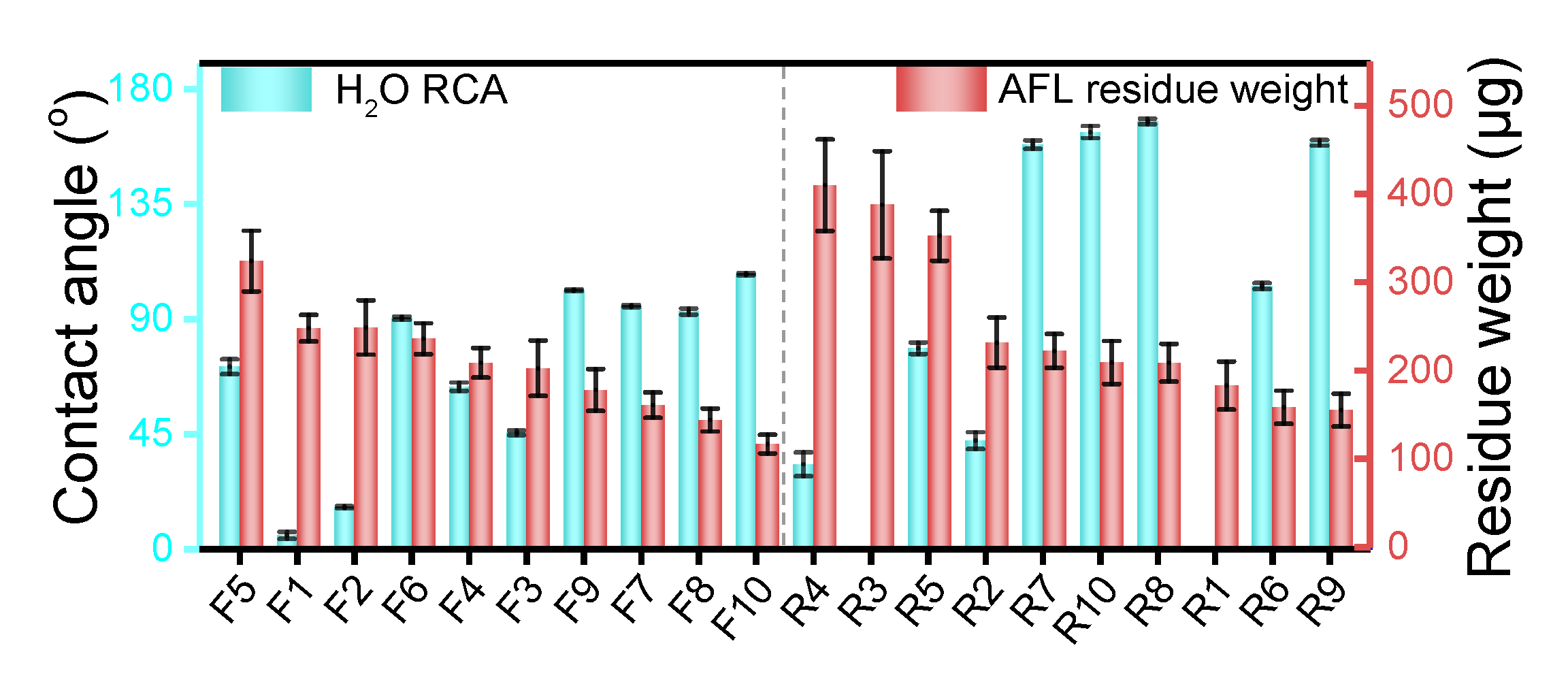


**Figure S4**. The relationship between the AFL residue weight and water RCAs for the ten flat samples and the ten rough samples under investigation.


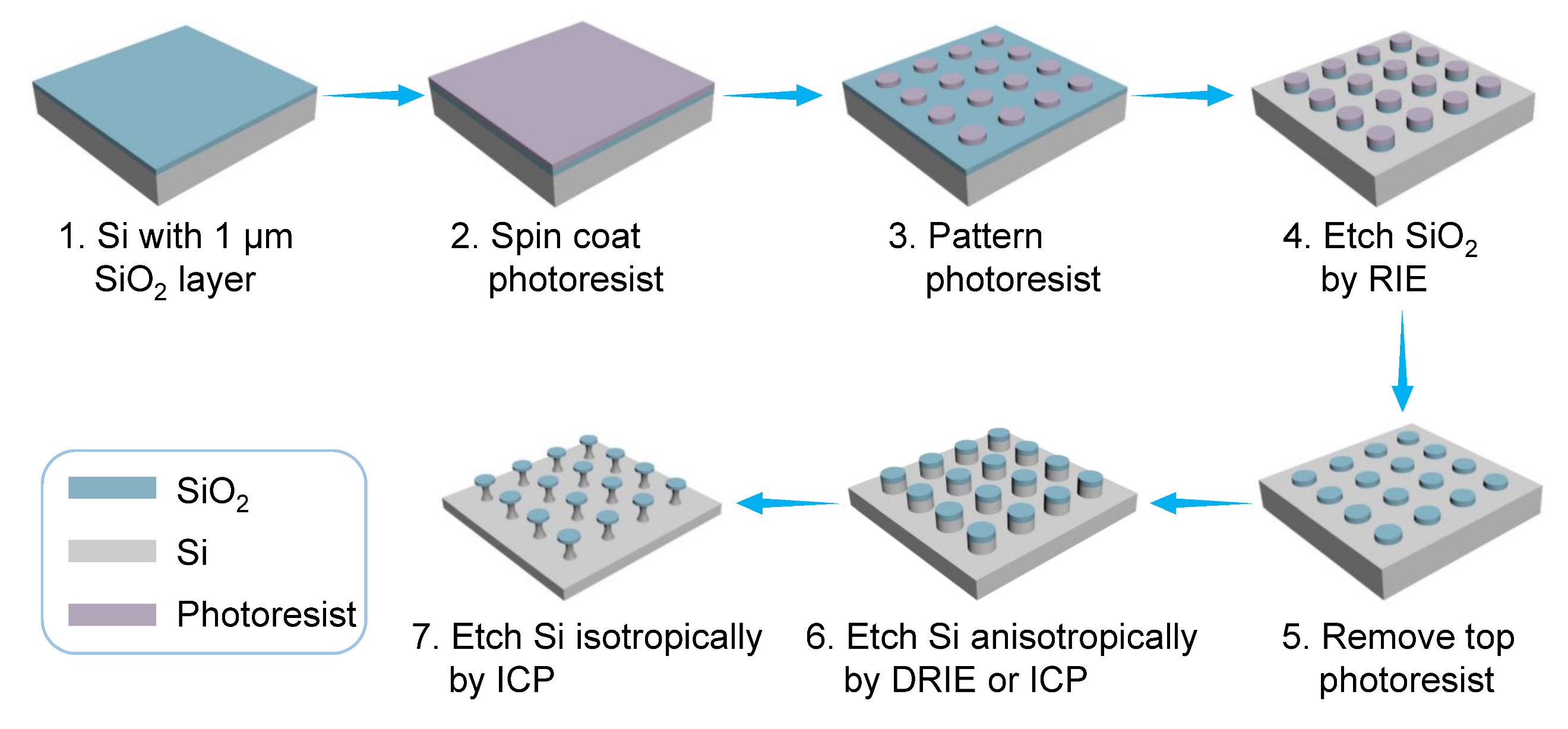


**Figure S5**. Schematic illustration of the microfabrication process of reentrant silicon post arrays.


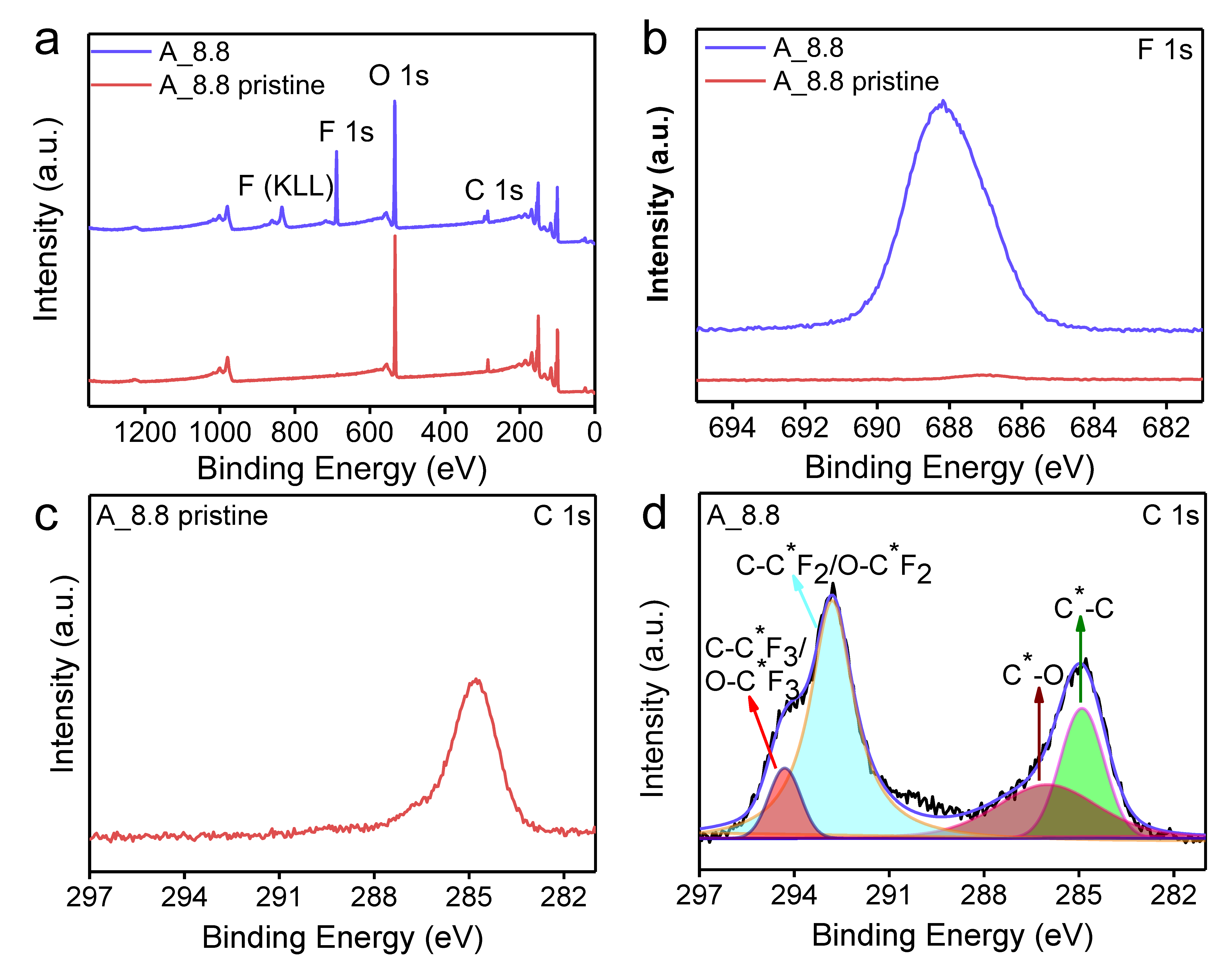


**Figure S6**. (a) The XPS spectra of the pristine and the PFPE-coated reentrant posts. XPS data of A_8.8 was used here as an example to demonstrate the successful grafting of PFPE. (b) F 1s spectra of the two samples. (c) C 1s spectrum of the pristine sample. (d) C 1s spectrum the PFPE-coated sample. The strengthened C signal and the appearance of F signal for A_8.8 indicate that PFPE was grafted to the surface. The C signal at 284.8 eV of the pristine sample might be associated with the residual photoresist with an atomic concentration of 6.39%. In comparison, after PFPE modification, C content reached to 14.67%. C^*^-O signal at 286.1 eV is due to the ether groups, and the peaks at 292.7 eV and 294.3 eV can be attributed to the abundant perfluoroalkylene (C-C^*^F_2_/O-C^*^F_2_) and perfluoroalkyl (C-C^*^F_3_/O-C^*^F_3_) domains of PFPE. The corresponding atomic concentrations are shown in Table S1.


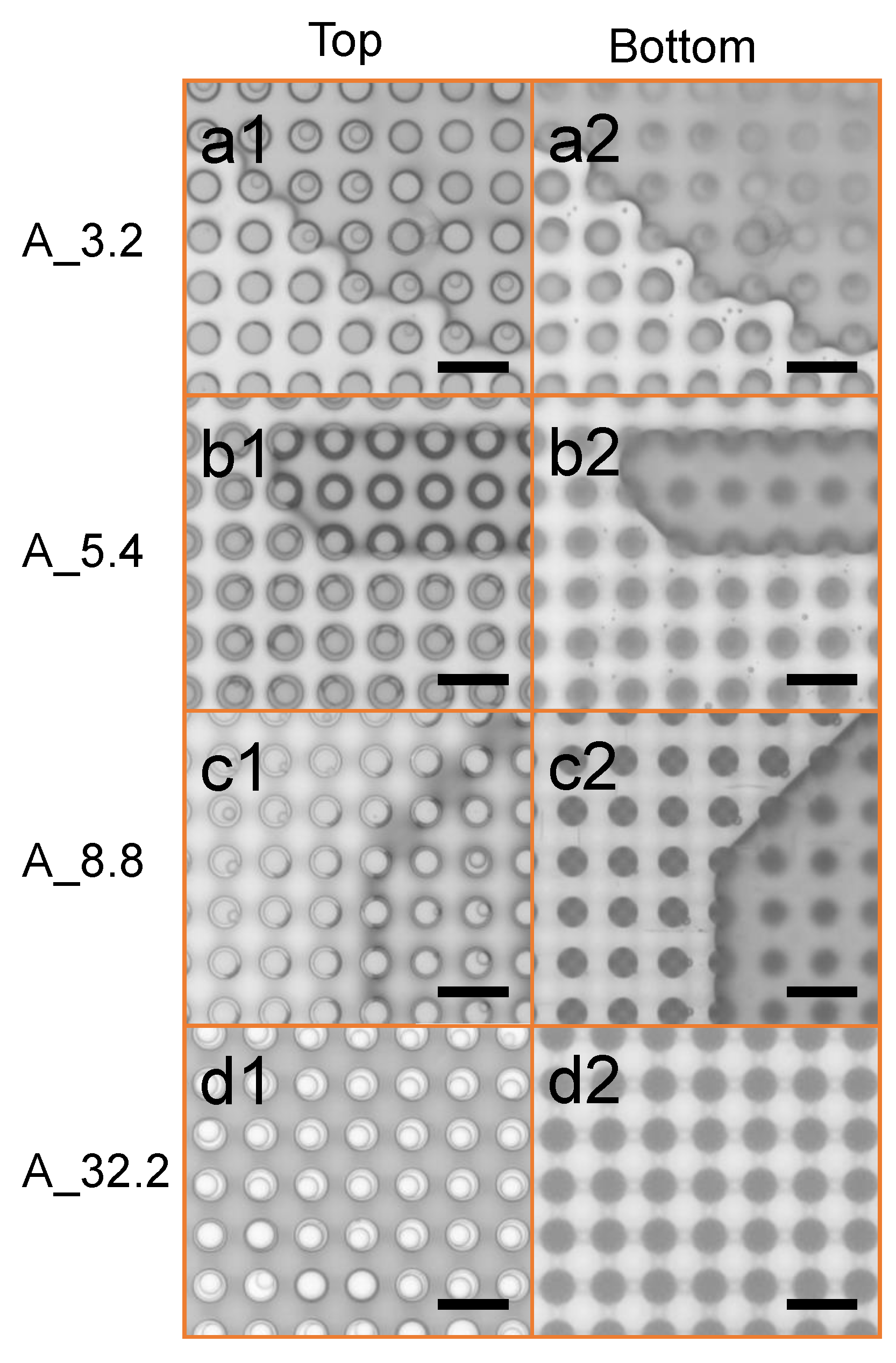


**Figure S7**. Optical microscope images showing the AFL residues on the reentrant post surfaces. The images were obtained by focusing on (a1-d1) the top plane and (a2-d2) the bottom plane of the reentrant post arrays. The residues were produced under a load of 200 g. AFL residues remained atop the posts of A_32.2 but collapsed into the textures of the other three samples. The scale bars are 20 μm.


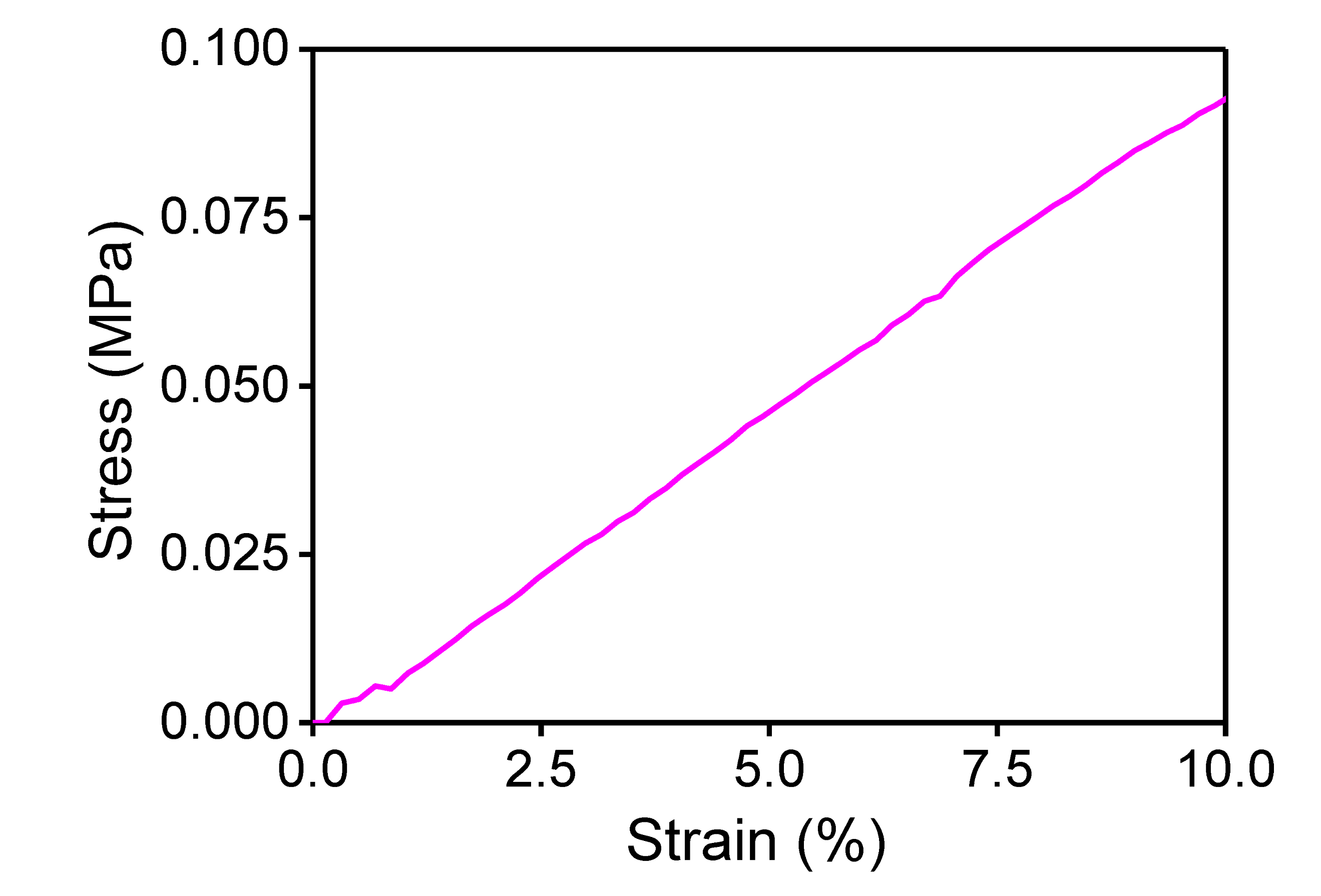


**Figure S8**. The tensile strain-stress plot of AFS. The elasticity modulus of AFS elastomer is calculated to be about 1 MPa.


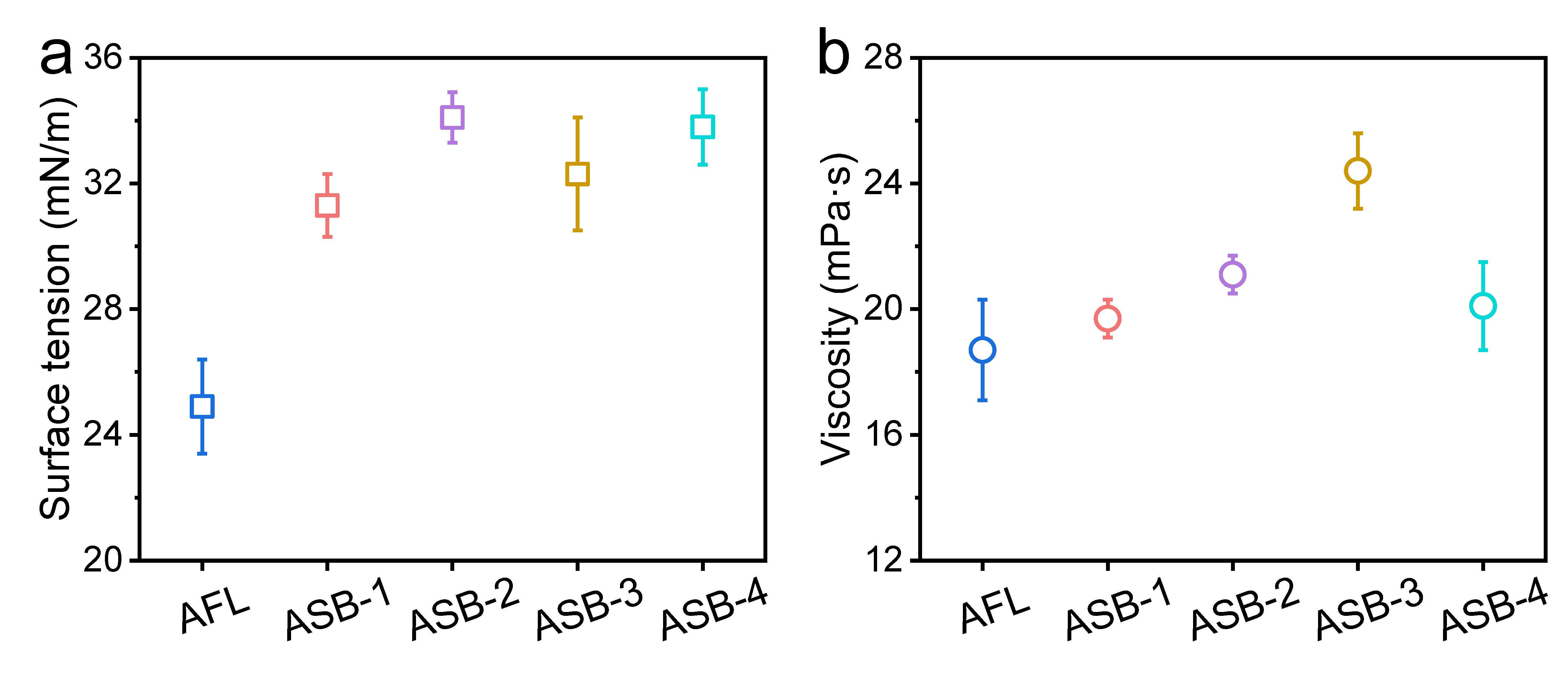


**Figure S9.** (a) Surface tension and (b) dynamic viscosity of AFL, ASB-1, ASB-2, ASB-3 and ASB-4, respectively.


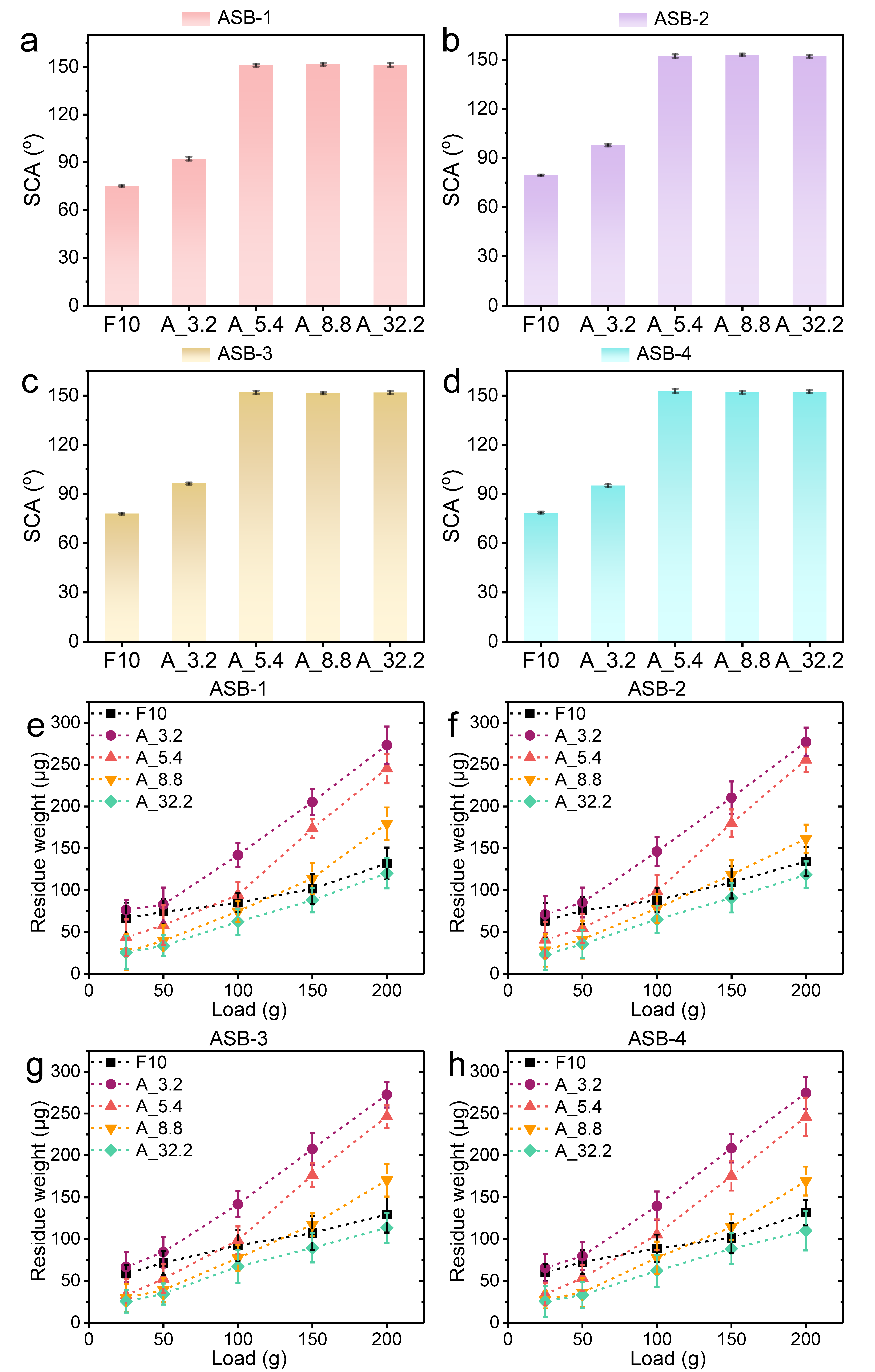


**Figure S10.** The SCAs of ASB-1 (a), ASB-2 (b), ASB-3 (c) and ASB-4 (d) on F10, A_3.2, A_5.4, A_8.8 and A_32.2, respectively. The relationship between the ASB residue weight and the loading pressure for different surfaces by using ASB-1 (e), ASB-2 (f), ASB-3 (g) and ASB-4 (h) as the probe liquids, respectively.


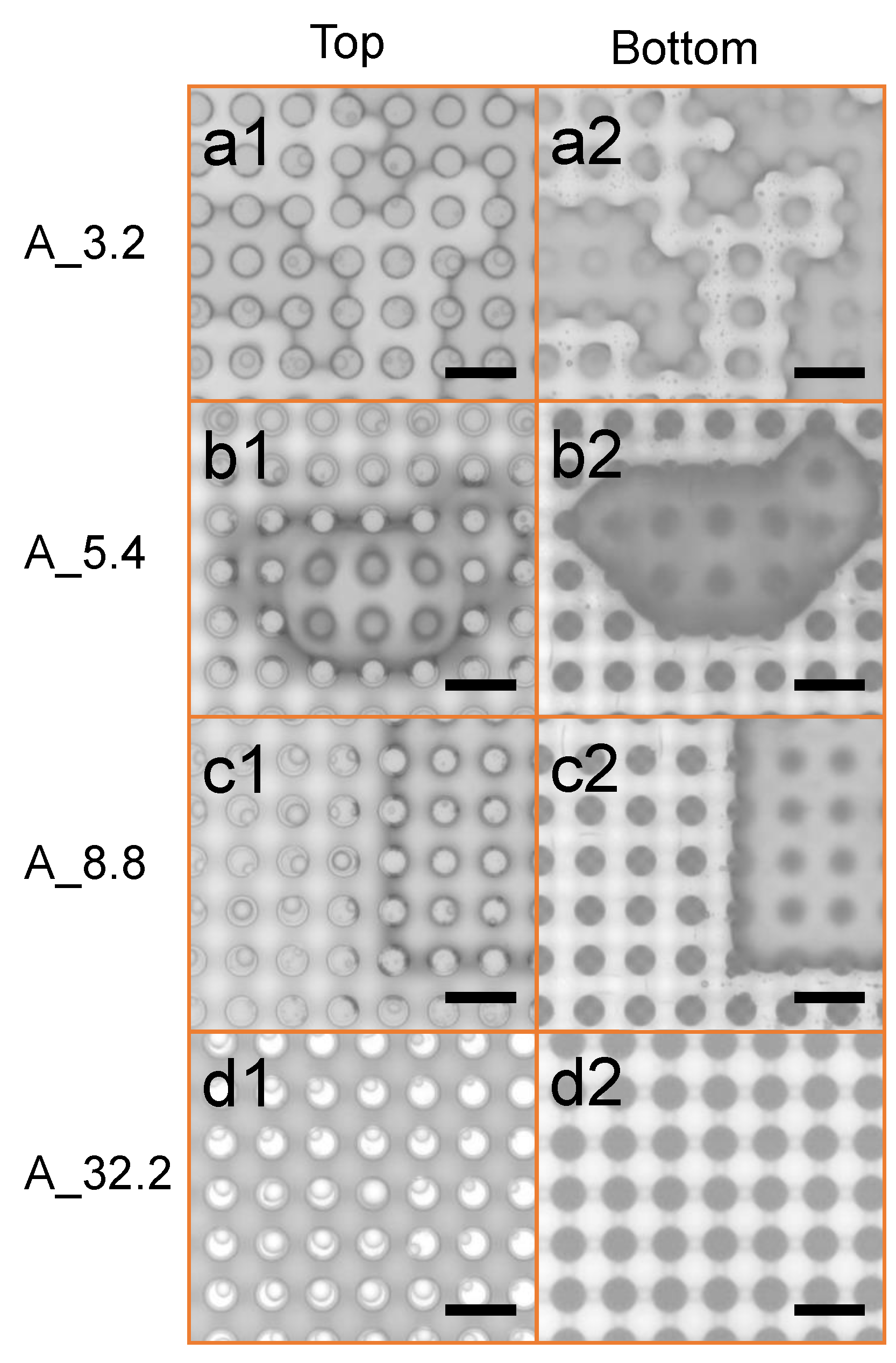


**Figure S11**. Optical microscope images showing the human fingerprint residues formed on the reentrant post surfaces. The images were obtained by focusing on (a1-d1) the top plane and (a2-d2) the bottom plane of the reentrant post arrays. The residues were produced by pressing the right index finger of an adult male (age 31) against the surfaces under a pressure of about 200 g. Fingerprint liquid was collected from his forehead to the finger before pressing. The test was conducted at an ambient humidity of 63% and a temperature of 24 °C in October (Guangzhou). It can be seen that the formation and wetting behaviors of human fingerprint residues are similar to those observed with AFL and AFS (Figure S7). The fingerprint residues remained on the top of the posts of A_32.2, but collapsed into the structures for the other three samples. The scale bars are 20 μm.


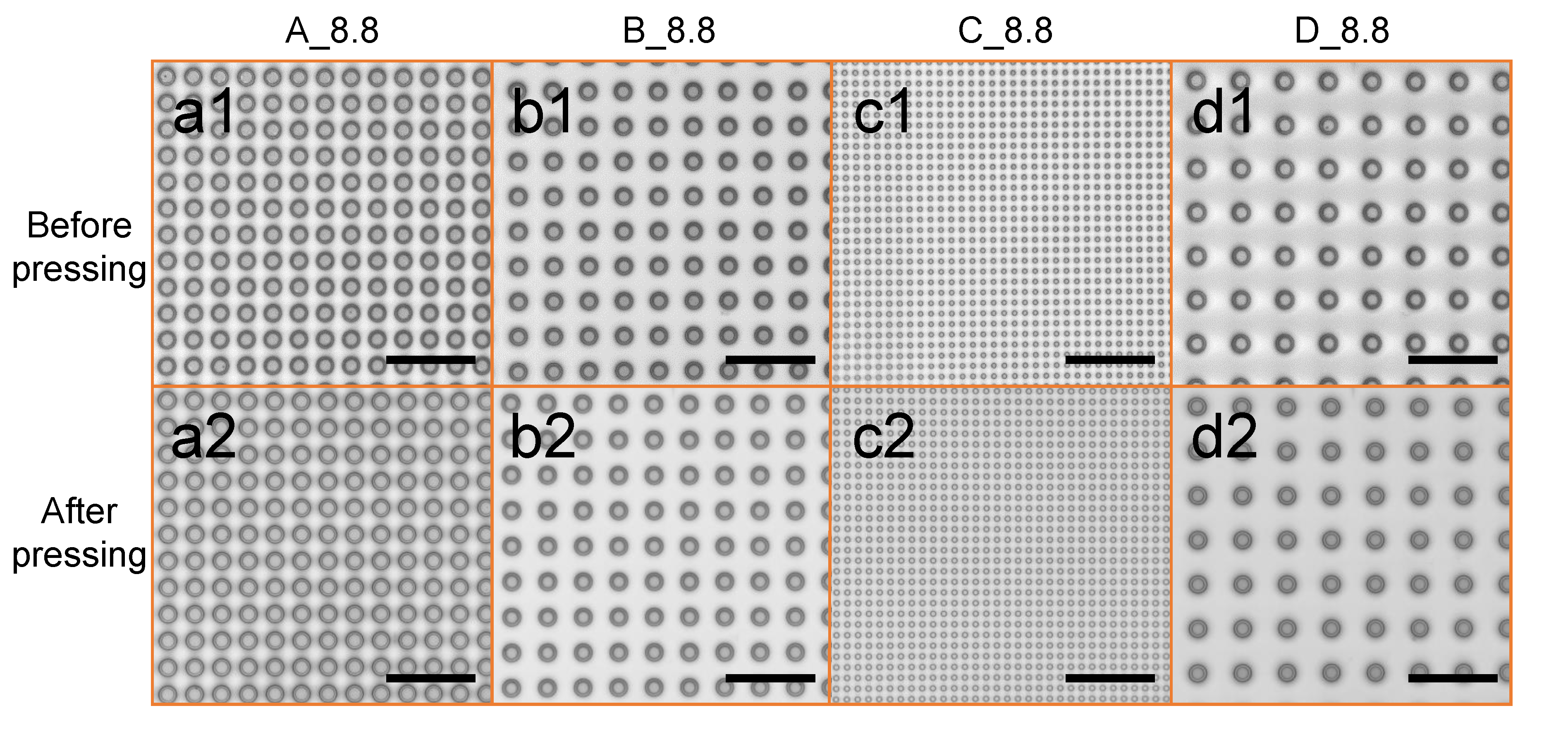


**Figure S12**. Optical microscope images showing the morphology of the reentrant post surfaces (including A_8.8, B_8.8, C_8.8 and D_8.8) before (a1-d1) and after (a2-d2) 300 pressings with 50 g load. The surfaces kept intact after pressing, demonstrating the durability of the posts. The scale bars are 50 μm.


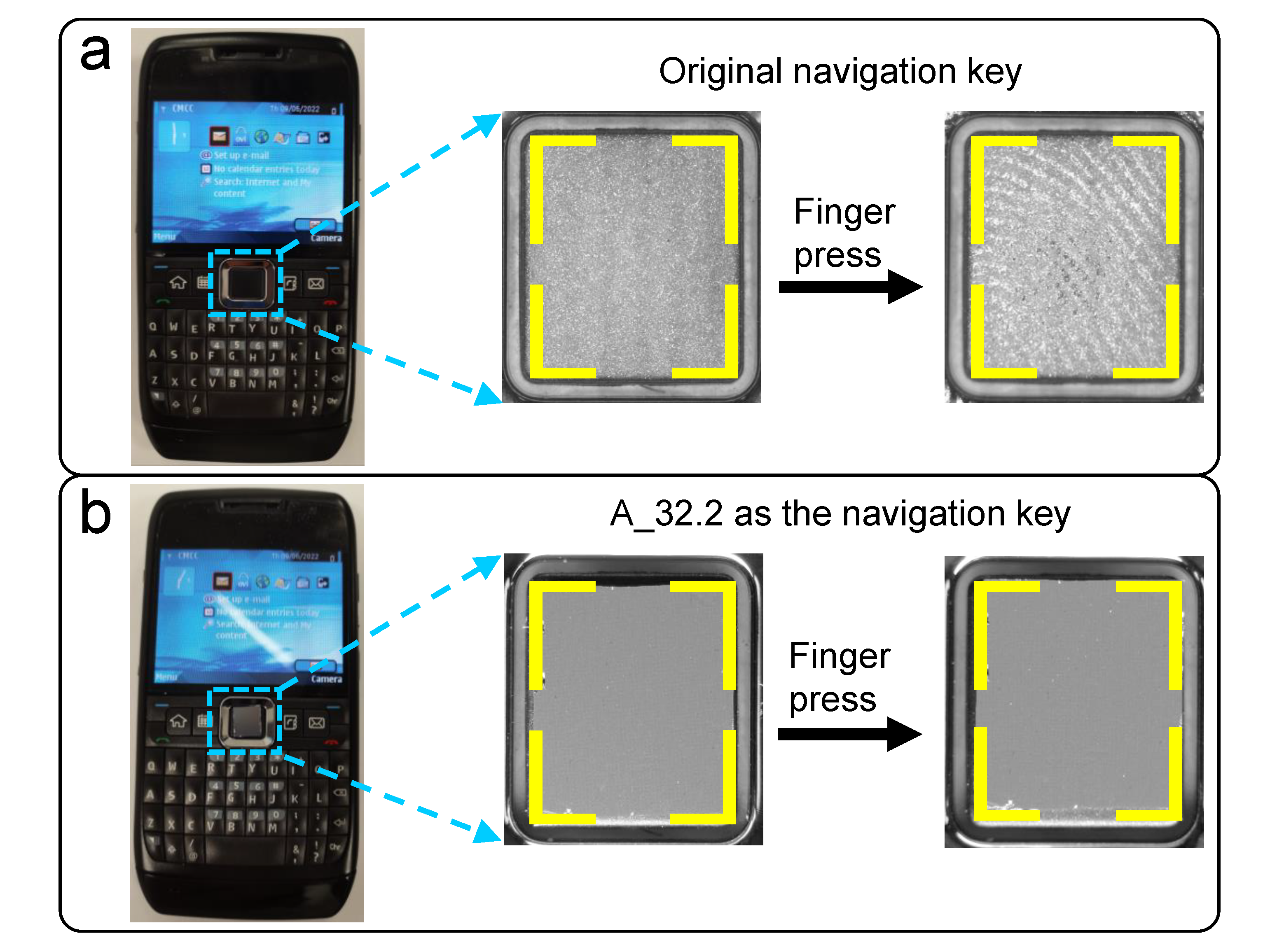


**Figure S13**. Demonstration of the application of A_32.2 as anti-fingerprint navigation key in consumer electronics. Anti-fingerprint test shows that (a) the original navigation key of a mobile phone was seriously contaminated by human fingerprint and (b) using A_32.2 as the navigation key could effectively prevent fingerprint contamination. The fingerprint was produced by the right index finger of an adult male. The pressing load was about 100 g.

**Table S1**. The atomic concentration (%) of the pristine and the PFPE-coated reentrant posts (A_8.8).

|  | Atomic concentration (%) | | | |
| --- | --- | --- | --- | --- |
|  | Si 2p | C 1s | O 1s | F 1s |
| A_8.8 pristine | 57.79 | 6.39 | 35.52 | 0.31 |
| A_8.8 | 37.28 | 14.67 | 30.87 | 17.19 |

**Table S2**. The structural parameters, AFL breakthrough pressures and robustness factors of the microtextured surfaces composed of reentrant posts, including A_3.2, A_5.4, A_8.8, A_32.2.

|  | A_3.2 | A_5.4 | A_8.8 | A_32.2 |
| --- | --- | --- | --- | --- |
| *2r*/μm | 10 | 10 | 10 | 10 |
| *d*/μm | 15 | 15 | 15 | 15 |
| *h*/μm | 3.2 | 5.4 | 8.8 | 32.2 |
| *f_sl_* | 0.35 | 0.35 | 0.35 | 0.35 |
| *P_crit_/*Pa | ^a)^ | 4571 | 4571 | 4571 |
| *RF* | ^a)^ | 161 | 161 | 161 |

^a)^ A_3.2 fails to show superoleophobicity towards AFL drops due to the sagging of liquid-gas interface, and the breakthrough pressure and RF are thus not shown.

**Table S3.** The formulation and references/source of the fingerprint-mimic liquids, namely AFL, ASB-1, ASB-2, ASB-3 and ASB-4.

| Liquids | Formulations | References/source |
| --- | --- | --- |
| AFL | 1-methoxy-2-propanol 20 wt%  Hydroxyl-terminated polydimethylsiloxane (Viscosity∼25 cSt) 20 wt%  Sodium chloride 10 g/L  Sodium hydrogen phosphate 10 g/L  Acetic acid 5 mL/L  Lactic acid 3 mL/L  Deionized water | [1-7] |
| ASB-1 | Triglyceride 59 wt%  Isostearate 24 wt%  Squalene 17 wt% | Commercial product |
| ASB-2 | Vegetable oil (Mazola) 45 wt%  Jojoba oil 25 wt%  Oleic acid 17 wt%  Squalene 13 wt% | [8-9] |
| ASB-3 | Triglyceride 40 wt%  Oleic acid 26 wt%  Wax ester 20 wt%  Squalene 10 wt%  Palmitic acid 2 wt%  Cholesterol 2 wt% | [10-11] |
| ASB-4 | Olive oil 20 wt%  Coconut oil 15 wt%  Synthetic spermaceti 15 wt%  Palmitic acid 10 wt%  Paraffin wax 10 wt%  Oleic acid 10 wt%  Stearic acid 5 wt%  Squalene 5 wt%  Cholesterol 5 wt%  Linoleic acid 5 wt% | [12] |

**Table S4**. The structural parameters of the microtextured surfaces composed of reentrant posts, including A_8.8, B_8.8, C_8.8 and D_8.8.

|  | A_8.8 | B_8.8 | C_8.8 | D_8.8 |
| --- | --- | --- | --- | --- |
| 2*r*/μm | 10.0 | 10.0 | 2.5 | 10.0 |
| *d*/μm | 15 | 20 | 6 | 25 |
| *h*/μm | 8.8 | 8.8 | 8.8 | 8.8 |
| *f_sl_* | 0.35 | 0.19 | 0.14 | 0.12 |
| *P_crit_/*Pa | 4571 | 2083 | 5387 | 1132 |
| *RF* | 161 | 73 | 174 | 40 |

**Table S5**. Calculation of the intrusion depth of AFS into the textures of reentrant surfaces with different *f_sl_* (Figure 5). The pressure-dependent contact area ratio was extracted from optical images using ImageJ. The AFS intrusion depths vary from 2.3 ± 0.4 μm to 5.6 ± 0.7 μm for the investigated samples, and the corresponding intrusion depths of AFL thus vary from 3.8 ± 0.4 μm to 7.1 ± 0.7 μm.

| Loading weight | Pressure  (*σ*) | Solid-liquid  contact fraction (*f_sl_*) | Pressure-dependent contact area ratio (*μ*) | AFS intrusion depth (Δ*l_s_*) |
| --- | --- | --- | --- | --- |
| 50 g | 1247 Pa | 0.35 (A_8.8) | 0.38 ± 0.07 | 2.3 ± 0.4 μm |
| 50 g | 1247 Pa | 0.19 (B_8.8) | 0.44 ± 0.03 | 3.6 ± 0.3 μm |
| 50 g | 1247 Pa | 0.14 (C_8.8) | 0.46 ± 0.03 | 4.7 ± 0.4 μm |
| 50 g | 1247 Pa | 0.12 (D_8.8) | 0.49 ± 0.06 | 5.6 ± 0.7 μm |

**Table S6**. The processing conditions of the microfabricated reentrant silicon post arrays.

| Samples | DRIE | ICP | |
| --- | --- | --- | --- |
|  |  | Anisotropic | Isotropic |
| A_3.2 | - | - | 1.5 min |
| A_5.4 | - | 1.5 min | 1.5 min |
| A_8.8 | 90 loops | - | 2 min |
| A_32.2 | 150 loops | - | 3 min |
| B_8.8 | 90 loops | - | 2 min |
| C_8.8 | 120 loops | - | 0.5 min |
| D_8.8 | 90 loops | - | 2 min |

**Movie S1, S2**

**Movie S1**. High-speed video shows the dynamic formation process of AFL residues on A_5.4, manifesting a “collapsed” state of the residues.

**Movie S2**. High-speed video shows the dynamic formation process of AFL residues on A_32.2, manifesting a “repellent” state of the residues.

**References**

[1] Wu, L. Y. L.; Ngian, S. K.; Chen, Z.; Xuan, D. T. T. Quantitative Test Method for Evaluation of Anti-Fingerprint Property of Coated Surfaces. *Appl. Surf. Sci.* **2011**, *257*, 2965-2969.

[2] Wang, G.; Wang, H.; Guo, Z. A Robust Transparent and Anti-Fingerprint Superhydrophobic Film. *Chem. Commun.* **2013**, *49*, 7310-7312.

[3] Rabnawaz, M.; Liu, G.; Hu, H. Fluorine-Free Anti-Smudge Polyurethane Coatings. *Angew. Chem., Int. Ed.* **2015**, *54*, 12722-12727.

[4] Rabnawaz, M.; Liu, G. Graft-Copolymer-Based Approach to Clear, Durable, and Anti-Smudge Polyurethane Coatings. *Angew. Chem., Int. Ed.* **2015**, *54*, 6516-6520.

[5] Zhong, X.; Hu, H.; Yang, L.; Sheng, J.; Fu, H. Robust Hyperbranched Polyester-Based Anti-Smudge Coatings for Self-Cleaning, Anti-Graffiti, and Chemical Shielding. *ACS Appl. Mater. Interfaces* **2019**, *11*, 14305-14312.

[6] Wu, X.; Liu, M.; Zhong, X.; Liu, G.; Wyman, I.; Wang, Z.; Wu, Y.; Yang, H.; Wang, J. Smooth Water-Based Antismudge Coatings for Various Substrates. *ACS Sustainable Chemistry & Engineering* **2017**, *5*, 2605-2613.

[7] Bender, D. N.; Zhang, K.; Wang, J.; Liu, G. Hard yet Flexible Transparent Omniphobic GPOSS Coatings Modified with Perfluorinated Agents. *ACS Applied Materials & Interfaces* **2021**, *13*, 10467-10479.

[8] Choi, H. J.; Park, K.-C.; Lee, H.; Crouzier, T.; Rubner, M. F.; Cohen, R. E.; Barbastathis, G.; McKinley, G. H. Superoleophilic Titania Nanoparticle Coatings with Fast Fingerprint Decomposition and High Transparency. *ACS Appl. Mater. Interfaces* **2017**, *9*, 8354-8360.

[9] Wertz, P. W. Human Synthetic Sebum Formulation and Stability Under Conditions of Use and Storage. *Int. J. Cosmetic Sci.* **2009**, *31*, 21-25.

[10] Cheng, J. B.; Russell, D. W. Mammalian Wax Biosynthesis. II. Expression Cloning of Wax Synthase cDNAs Encoding A Member of the Acyltransferase Enzyme Family. *J. Biol. Chem.* **2004**, *279*, 37798-37807.

[11] Stefaniak, A. B.; Harvey, C. J.; Wertz, P. W. Formulation and Stability of a Novel Artificial Sebum Under Conditions of Storage and Use. *Int. J. Cosmet. Sci.* **2010**, *32*, 347-355.

[12] ASTM D4265-14. Standard Guide for Evaluating Stain Removal Performance in Home Laundering. ASTM International, West Conshohocken, PA, USA, **2014**.
